# Glutathione Promotes Degradation and Metabolism of Residual Fungicides by Inducing UDP-Glycosyltransferase Genes in Tomato

**DOI:** 10.3389/fpls.2022.893508

**Published:** 2022-07-01

**Authors:** Gaobo Yu, Qiusen Chen, Fengqiong Chen, Hanlin Liu, Jiaxin Lin, Runan Chen, Chunyuan Ren, Jinpeng Wei, Yuxian Zhang, Fengjun Yang, Yunyan Sheng

**Affiliations:** ^1^College of Horticulture and Landscape Architecture, Heilongjiang Bayi Agricultural University, Daqing, China; ^2^College of Agriculture, Heilongjiang Bayi Agricultural University, Daqing, China; ^3^College of Tropical Crop, Hainan University, Haikou, China; ^4^Ministry of Agriculture and Rural Affairs Agro-products and Processed Products Quality Supervision, Inspection and Testing Center, Daqing, China

**Keywords:** UDP-glycosyltransferase, tomato (*Solanum lycopersicum*), glutathione, chlorothalonil residues, gene expression

## Abstract

Reduced glutathione (GSH) is a key antioxidant, which plays a crucial role in the detoxification of xenobiotics in plants. In the present study, glutathione could reduce chlorothalonil (CHT) residues in tomatoes by inducing the expression of the UDP-glycosyltransferase (UGT) gene. In plants, UGT is an important glycosylation catalyst, which can respond to stresses in time by activating plant hormones and defense compounds. Given the importance of plant growth and development, the genome-wipe analyses of Arabidopsis and soybean samples have been carried out, though not on the tomato, which is a vital vegetable crop. In this study, we identified 143 UGT genes in the tomato that were unevenly distributed on 12 chromosomes and divided into 16 subgroups and found that a variety of plant hormones and stress response cis-elements were discovered in the promoter region of the SlUGT genes, indicating that the UGT genes were involved in several aspects of the tomato stress response. Transcriptome analysis and results of qRT-PCR showed that most SlUGT genes could be induced by CHT, and the expression of these genes was regulated by glutathione. In addition, we found that SlUGT genes could participate in plant detoxification through interaction with transcription factors. These findings further clarify the potential function of the UGT gene family in the detoxification of exogenous substances in tomatoes and provide valuable information for the future study of functional genomics of tomatoes.

## Introduction

In modern agriculture, pesticides have become an important factor to prevent crop losses and increase yield. However, excessive exposure to pesticides can lead to disruption of endocrine, mitochondrial (dys) function, and genetic and epigenetic modifications (Mostafalou and Abdollahi, 2013). Pesticides, therefore, pose a direct threat to human health. Moreover, they are phytotoxic to plants, potentially inhibiting plant growth, photosynthesis, and the synthesis of proteins, DNA, and pigments (Liu et al., 2021; Shahid et al., 2021). In addition, it is worth noting that pesticide residues may remain in plant food organs. Pesticide residues, seriously affect food safety and crop quality and even threaten human health and the environment, which is a global problem (Carvalho, 2017). Plant have effective antioxidant mechanisms to respond to the adverse environments and toxicity of xenobiotics (Peco et al., 2020). At the same time, plants could utilize a three-phase detoxification system to transform pesticides, which typically involves conversion, conjugation, and compartmentalization processes (Su et al., 2019). In the first stage, pesticides are transformed into less toxic products by catalyzing oxidation, reduction, and hydrolysis catalyzed through cytochrome P450 monooxygenase and peroxidase. The second stage involves coupling pesticide metabolites to sugars, amino acids, or glutathione by glutathione-S-transferase (GST) and uridine diphosphate UDP-glycosyltransferase (UGT), which is less toxic and more soluble in water. In the third stage, these metabolites are transported from the cytoplasm to the vacuoles or blastocysts in plants (Sun et al., 2018). Glutathione (GSH) is a small peptide consisting of three amino acids, which is an important antioxidant and free radical scavenger in plants. At the same time, it can be used as the substrate of GST to participate in the two-phase detoxification in plants, to effectively alleviate the stress of toxic xenobiotics (Geu-Flores et al., 2011).

UGT is an enzyme that catalyzes glycosylation, which generally catalyzes the transfer of the glycosyl group from nucleoside diphosphate-activated sugars to a wide range of substrates (Barvkar et al., 2012). It exists widely in plants and plays an important role in response to abiotic stress (Chen T. et al., 2020). The plant UGT has a conserved sequence of 44 amino acids at the C-terminus, which is the plant’s secondary product glycosyltransferase box (PSPG box), the PSPG box is responsible for glycosyl binding (Caputi et al., 2012). N-terminal sequences vary widely and are responsible for identifying different receptor molecules (Vogt and Jones, 2000).

The UGT has been extensively studied in various plant species. In *Arabidopsis thaliana*, *Zea mays*, *Glycine max*, and *Citrus grandis*, over 100UGTs have been identified and classified into 14–17 groups based on the conservation of their amino acid sequences (Li et al., 2001, 2014; Mamoon Rehman et al., 2016; Wu et al., 2020). The functions of many UGT genes have been identified in plants and are related to different pathways. In particular, great progress has been made in the study of stress-related UGT. For example, *AtUGT73B3* and *AtUGT73B5* could respond to pathogens, thereby increasing the resistance of Arabidopsis to biotic stress (Langlois-Meurinne et al., 2005). Overexpression of *UGT85A5* could effectively enhance the salt tolerance of tobacco (Sun et al., 2013). *UGT91Q2* in tea plants plays a key role in regulating cold tolerance (Zhao et al., 2020).

Although the UGT gene plays an important role in the resistance of many plants to abiotic stresses, the UGT gene family in tomatoes is still not well understood. Therefore, in this study, genome-wide identification of the UGT gene family of tomato was characterized. The potential function of the UGT gene family in response to abiotic stresses in tomatoes was further clarified by phylogenetic relationship, chromosome location, expression profile, and protein structure analysis.

## Materials and Methods

### Plant Materials and Treatments

The seeds of the tomato cultivar “Zheza205” were grown on mixed soil in pots. Once six true leaves were fully expanded, the plants were pretreated with ddH_2_O, 1 mM BSO, or 5 mM GSSG, respectively, and then sprayed with 11.2 mM CHT after 24 h of pretreatment. The leaves were sampled at 24 h after CHT treatment for physiological indexes and qRT-PCR analysis, while the samples were taken on the 7th day after CHT treatment for the determination of pesticide residues. Triplicates were maintained for each treatment and control sample. The samples were immediately frozen in liquid nitrogen and stored at −80°C until further analyses.

### Determination of CHT Residues in Plants

The tomato sample (5 g) was grounded with 20 ml methanol. The extracted supernatant was used for analysis by UPLC. The residue of CHT in tomato was determined according to the method of Jongen et al. (1991).

The content of NPT in tomato was determined according to the method of Pal et al. (2019). The 0.2 g sample was homogenized in 3 ml of 5-sulfosalicylic acid (6.67%) and centrifuged at 13,000 × *g* at 4°C for 10 min. Diluted with Ellman reagent (1:9), the supernatant was taken after 15 min and the absorbance was recorded at 412 nm.

### Analysis of Antioxidant Enzymes Activities

One gram (FW) tomato leaves were homogenized in 5 ml of 100 mM sodium phosphate buffer (pH 7.5) to determine the antioxidant enzymes. The determination of superoxide dismutase (SOD) activity by measuring the inhibition of NBT reduction at 560 nm (Beauchamp and Fridovich, 1971). The determination of catalase (CAT) activity by monitoring the decomposition of H_2_O_2_ at 240 nm (Aebi, 1984). The determination of ascorbate peroxidase (APX) activity was measured by spectrophotometric monitoring is based on ascorbic acid oxidation (Nakano and Asada, 1981). The determination of glutathione peroxidase (GPX) activity was measured by monitoring the non-enzymatic oxidation of NADPH (Halliwell and Foyer, 1978).

### Analysis of Non-enzymatic Substances

The content of ascorbic acid (AsA) in tomato leaves was estimated by homogenizing 0.5 g of sample tissue at 5% (w/v) metaphosphoric acid (Mukherjee and Choudhuri, 1983). The reduction level glutathione (GSH) in tomato leaves was estimated by homogenizing fresh sample tissue in 5% (w/v) of sulpho-salicylic acid (Smith, 1985).

### Analysis of ROS Content

The method of Sinha (1972) was used to determine the concentration of hydrogen peroxide (H_2_O_2_) in tomato leaves. Homogenizing 0.2 g of tomato leaves in ice-cold 0.1 M of PBS (pH 7.0). After centrifugation, the absorbance value was measured at 570 nm based on H_2_O_2_ mediated Fe^2+^ oxidation. According to the method of Chen et al. (2019), 0.2 g fresh leaves were homogenized in ice-cold 50 mM of PBS (pH 7.8) for the determination of superoxide radicals (O_2_^−^) concentration in tomato leaves.

### Identification of the SlUGTs Family

One hundred twelve known IDs of *A. thaliana* UGT genes were put into the Arabidopsis genome database (TAIR)^1^ for protein sequences. Using these Arabidopsis UGT protein sequences as probes, the 146 candidate members of the *Solanum lycopersicum* UGT family were searched using BLASTP. On the SMART database,^2^ the domain of Pfam (PF00201) was identified and screened in the phytozome database. Subsequently, the duplicate UGT gene was deleted by DNAMAN (version 7.212, Lynnon Corp., Quebec, Canada), and genes with related amino acid sequence <200 were removed. Finally, 143 SlUGT genes were obtained and named *SlUGT1–SlUGT143* according to their chromosomal positions. The grand average of hydropathicity (GRAVY), the number of amino acids, theoretical isoelectric points (pI), and secondary structure of all predicted SlUGTs were then determined by ExPASy, and predicting the subcellular localization using Plant-mPLoc (Wilkins et al., 1999; Chou and Shen, 2010).

### Phylogenetic Analysis and Multiple Sequence Alignment

The SlUGT protein sequence were obtained from the NCBI protein database.^3^ Use Gblocks^4^ to remove fuzzy areas in the alignment, and use conserved areas to construct phylogenetic trees (Castresana, 2000). Use MUSCLE to create multiple sequence alignment of all SlUGTs (Zhang et al., 2021). A neighbor-joining (NJ) phylogenetic tree of full-length sequences of AtUGTs and SlUGTs was constructed with 1,000 bootstrap replicates, using MEGA 7.0 (Morgulis et al., 2008). Identification of putative homologous chromosomal regions between tomato genome, *Zea* genome, and *A. thaliana* genomes genome using MCScanX toolkit, and then visualized with TBtools (Yu et al., 2017).

### Gene Structure, Conserved Domain, and Motif Analysis of SlUGT Genes

The Gene Structure Display Server (GSDS v2.0)^5^ is used to determine the exon-intron sequences of SlUGT genes by comparing the coding sequence of each SlUGT gene with its genome sequence (Hu et al., 2015). Search for the novel motifs of SlUGTs using MEME 5.0.3^6^ (Bailey et al., 2009).

### Identification of Plant Growth Regulator-Related cis-Elements

To identify the potential cis-acting sequences responsible for stress response in the promoter region of the UGTs, the cis-elements of SlUGT promoter regions were detected using the plant cis-acting regulatory element (Plant CARE) database with the default parameters (Rombauts et al., 1999). The promoter region of SlUGT is the genomic DNA sequence of 2000 bp upstream of the initiation codon (ATG).

### Expression Profiles of SlUGT Genes in Diverse Tissues

UGT genes expression data in different tissues were obtained from the online tomato eFP database.

### Analysis of the Expression Levels of SlUGT Genes From Transcriptome Data

Tomato seedlings with six leaves were pretreated with ddH_2_O, 1 mM BSO, or 5 mM GSSG, respectively, after 24 h pretreatment, which was treated with 11.2 mM of chlorothalonil (CHT). After 24 h exposure to CHT, the leaves were sampled and analyzed by transcriptome sequencing. The expression profiles of the differentially expressed genes were presented. Amazing HeatMap software (Chen C. et al., 2020) was used to generate a heatmap.

### RNA Extraction and qRT-PCR Assays

TRIzol® reagent (Invitrogen, Carlsbad, CA, United States) were used to extract total RNA. One microgram per RNA sample was used as the template for synthesis of the first-strand cDNA, using the ReverTra Ace™ qPCR RT Master Mix with gDNA Remover (TOYOBO Co., Osaka, Japan). Next, using SYBR® Select Master Mix RT-PCR System (Takara) performed qRT-PCR on an optical 96-well plate. Actin was used as an internal reference. All the primers used for gene expression analysis were shown in Supplementary Table S1. The relative expression level was calculated with the formula 2^−ΔΔCT^ (Livak and Schmittgen, 2001; Cui et al., 2019). Three independent biological replicates were analyzed.

### Prediction of Transcription Factors of Tomato UGT Gene Family

We used PlantRegMap (Internet; Tian et al., 2020)^7^ to predict transcription factors associated with SlUGT. Meanwhile, the transcription factors (TFs) network was visualized using Origin 8.0.

## Results

### Effect of Exogenous Treatment on Chlorothalonil Residue in Tomato

**Figure 1:**
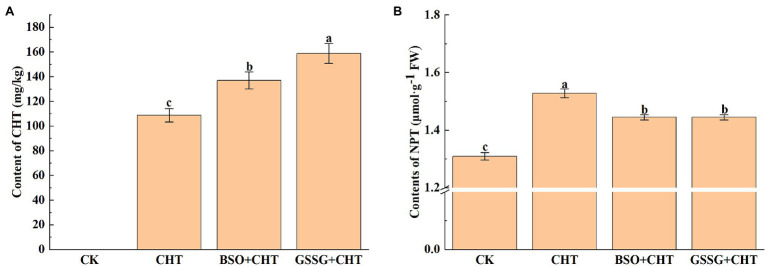
Pesticide residue **(A)** and NPT content **(B)** under exogenous treatment in tomato. NPT, non-protein thiols; CHT, chlorothalonil; BSO, L-buthionine-sulfoximine; and GSSG, glutathione (oxidized). Error bars represented the SD (*n* = 3). According to Duncan’s multiple test, bars with different letters were significantly different (*p* < 0.05).

To evaluate the effect of glutathione on CHT accumulation in tomato leaves, the content of CHT in the leaves of tomatoes was analyzed under different treatments. Significant CHT accumulation was observed in the leaves of plants treated with CHT (Figure 1A). Interestingly, the application of BSO (Glutathione synthesis inhibitor) or GSSG on CHT-treated plants significantly increased the content of CHT in leaves by 26.0% and 46.1%, respectively, compared with that in only CHT-treated plants. In addition, we observed that exposure to CHT significantly increased the NPT content in leaves by 16.8%, while NPT content was inhibited with the application of BSO and GSSG (Figure 1B), suggesting that glutathione improved CHT detoxification in the tomato plant.

### Effects of Exogenous Treatment on Antioxidant System in Tomato

**Figure 2:**
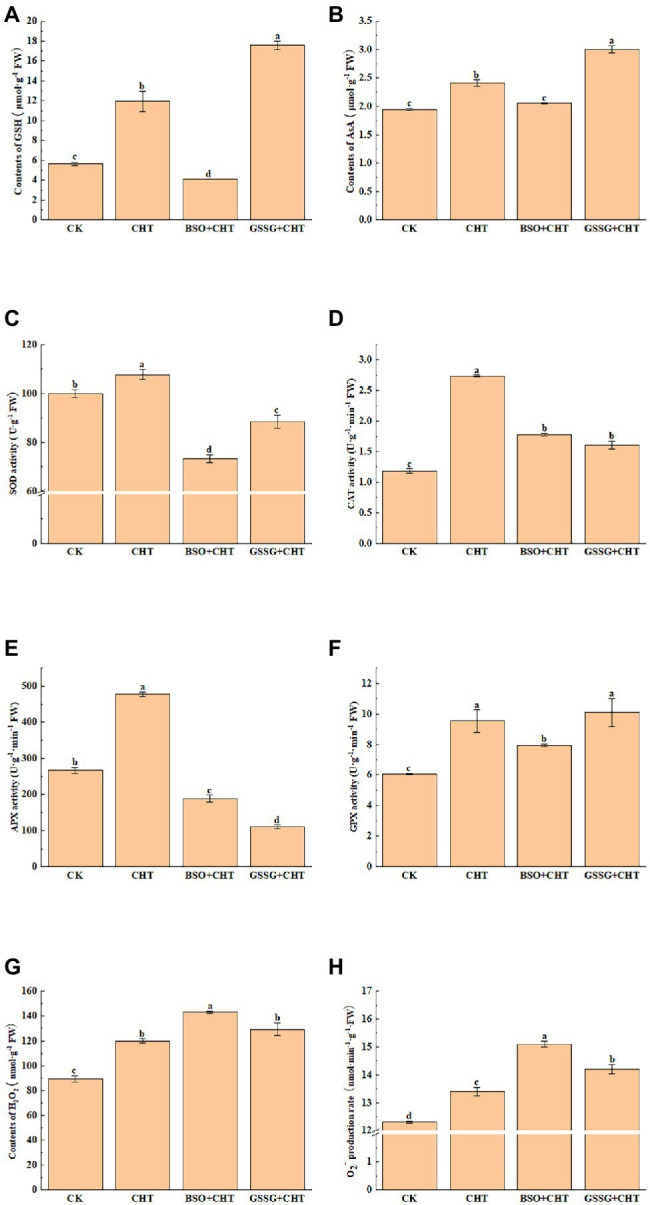
Effects of exogenous treatment on antioxidant system in tomato. **(A)** GSH content. **(B)** AsA content. **(C)** SOD activity. **(D)** CAT activity. **(E)** APX activity. **(F)** GPX activity. **(G)** H_2_O_2_ content. **(H)** O_2_^−^ content. GSH, glutathione; AsA, ascorbic acid; SOD, superoxide dismutase; CAT, catalase; APX, ascorbate peroxidase; H_2_O_2_, hydrogen peroxide; O_2_^−^, superoxide anion; CHT, chlorothalonil; BSO, L-buthionine-sulfoximine; and GSSG, glutathione (oxidized). Error bars represented the SD (*n* = 3). According to Duncan’s multiple test, bars with different letters were significantly different (*p* < 0.05).

To assess the CHT-caused injury to tomatoes, we analyzed ROS accumulation in tomatoes. The biochemical assay of ROS showed that CHT treatment significantly increased the H_2_O_2_ and O_2_^−^ levels by 34.4% and 6.3%, respectively, compared to CK (Figures 2A,B). Meanwhile, a further increase was observed in the level of both H_2_O_2_ and O_2_^−^ in tomatoes under CHT stress with BSO application, while only an increase in the content of O_2_^−^ was found in tomatoes with GSSG treatment and no significant change in the production of H_2_O_2_.

To unveil how glutathione alleviates CHT-induced oxidative stress, we measured the activity of antioxidant enzymes such as SOD, CAT, APX, and GPX. The CHT treatment significantly increased the activity of SOD, CAT, APX, and GPX by 7.8%, 30.3%, 79.2%, and 57.7%, respectively, compared with the control (Figures 2C–F). However, BSO administration substantially inhibited the activity of SOD, CAT, APX, and GPX by 32.0%, 35.0%, 60.5%, and 16.9%, respectively, compared with the only CHT treatment. Similarly, GSSG treatment also decreased the activity of antioxidant enzymes except for GPX, compared with the only CHT treatment.

To evaluate the remediation role of glutathione in ROS stress, we also investigated the non-enzymatic substances exposed to CHT. AsA and GSH were two common plant antioxidants. The CHT treatment significantly promoted the content of AsA and GSH by 23.6% and 111.0%, respectively, compared with the CK (Figures 2G,H). However, the application of BSO on CHT-treated plants significantly decreased the content of AsA and GSH in leaves by 26.0% and 46.1%, respectively, compared with that in only CHT-treated plants. These results suggested that glutathione alleviated CHT-induced oxidative stress by the antioxidant enzymes and non-enzymatic substances in tomatoes. Interestingly, GSSG application increased the levels of GSH and AsA, compared with that in only CHT-treated plants. This might be caused by the change in glutathione redox homeostasis.

### Effects of Exogenous Treatment on Detoxification System in Tomato

**Figure 3:**
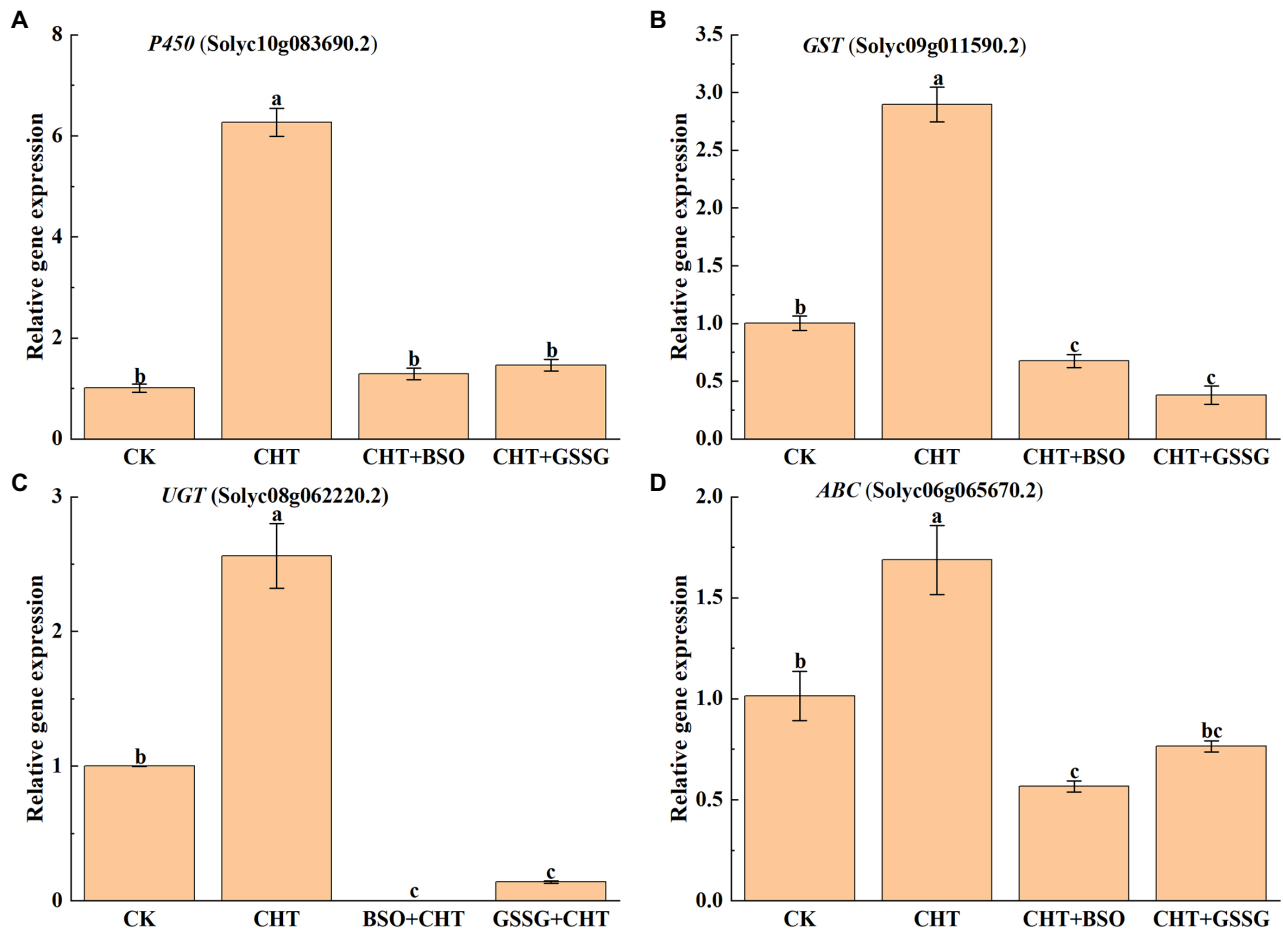
Effects of exogenous treatment on genes’ expression related to the detoxification system in tomato. **(A)** P450 expression. **(B)** GST expression. **(C)** UGT expression. **(D)** ABC expression. P450, cytochrome P450s; GST, glutathione S-transferase; UGT, UDP-glycosyltransferases; ABC, ATP-binding cassette transporters; CHT, chlorothalonil; BSO, L-buthionine-sulfoximine; and GSSG, glutathione (oxidized). Error bars represented the SD (*n* = 3). According to Duncan’s multiple test, bars with different letters were significantly different (*p* < 0.05).

P450, GST, UGT, and ABC play an important role in xenobiotic detoxification in plants. To further study the effect of glutathione on CHT detoxification as influenced, the gene expression of P450, GST, UGT and ABC were evaluated in tomato leaves. The results showed that the expression of P450, GST, UGT, and ABC increased after CHT treatment compared with the control. However, BSO administration substantially inhibited the expression of all these genes compared with the only CHT treatment. Similarly, GSSG treatment also reduced the expression of all these genes compared with the only CHT treatment (Figure 3), suggesting a role for glutathione in CHT detoxification in tomatoes.

### Identification and Chromosome Distribution of UGT Gene Family in Tomato

**Figure 4:**
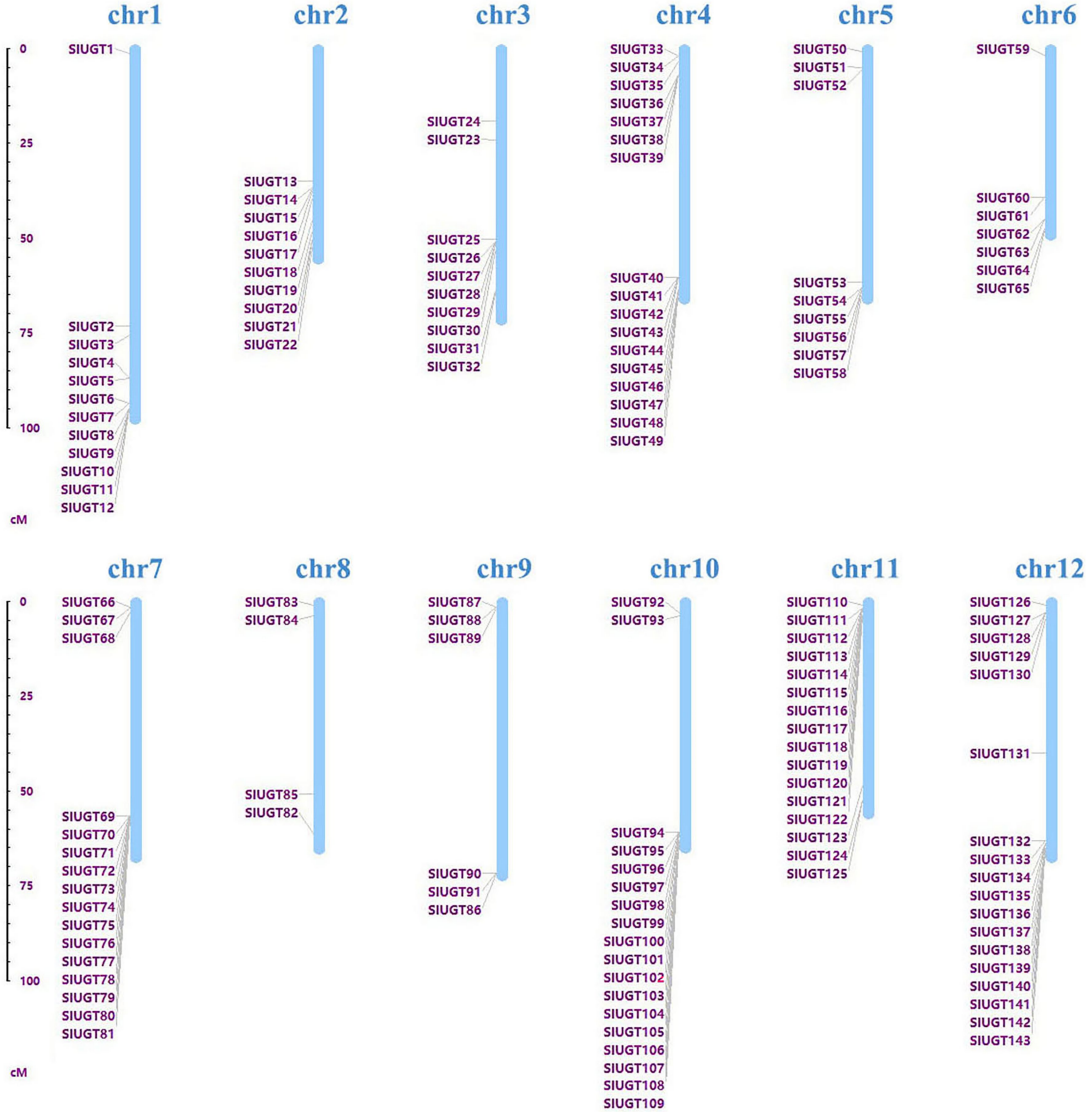
Chromosomal distribution of the 143 SlUGT genes identified in the present study. The chromosome number is indicated above each chromosome.

Taking the *A. thaliana* UGT protein sequence as a reference, the candidate UGT protein was screened through a blast comparison of the tomato genome [SL2.50 (iTAG2.4)]. One hundred forty-three tomato UGT genes were screened for the conserved domain (PF00201) and de-redundancy. According to the position of the gene on the chromosome, the candidate gene was named *SlUGT1 ~ SlUGT143* (Figure 4). The SlUGT protein sequence consisted of 292–952 amino acids, with an average length of 463 amino acids. Among them, 134 SlUGT proteins were acidic (with an isoelectric point <7), and only nine SlUGT proteins were alkaline (isoelectric point >7). The subcellular localization, as estimated by Plant-mPLoc, showed that SlUGT proteins were likely to be distributed in multiple organelles, including the peroxisome, mitochondria, and endoplasmic reticulum, though most SlUGTs were located in the chloroplast, cytoplasm, and nucleus. The total hydrophilic score of most SlUGT genes was negative, only three UGT genes (*SlUGT29*, *SlUGT35*, and *SlUGT51*) were positive in hydrophilicity, indicating that most SlUGT genes were hydrophilic (Supplementary Table S1).

### Phylogenetic Relationship of SlUGT Genes and Synteny Analysis of SlUGT Genes

**Figure 5:**
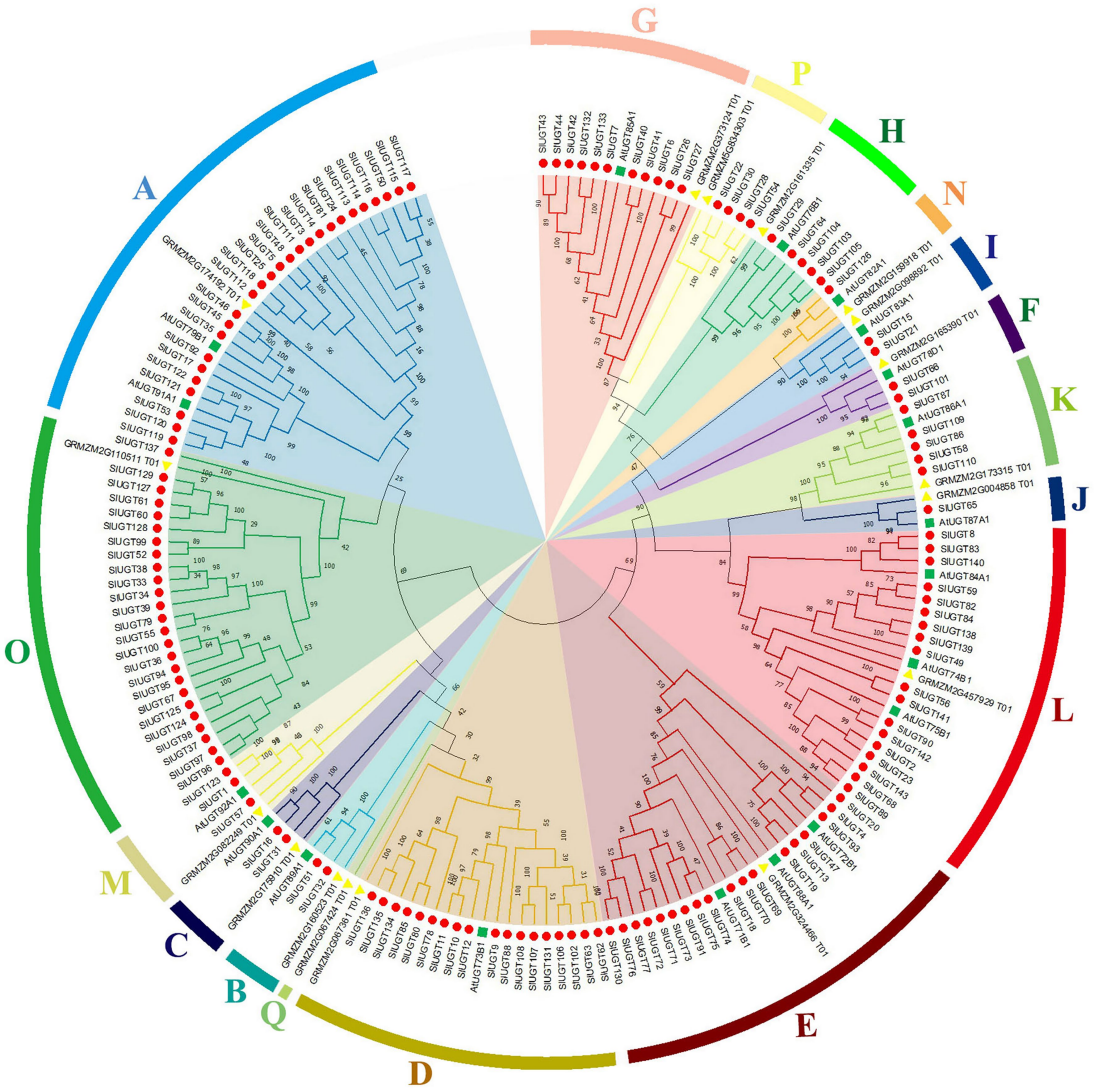
Phylogenetic analysis of UGT family genes from tomato. The tree was constructed using the neighbor-joining method by aligning the amino acid sequences of tomato UGTs, *Zea mays* UGTs, and *Arabidopsis* UGTs.

**Figure 6:**
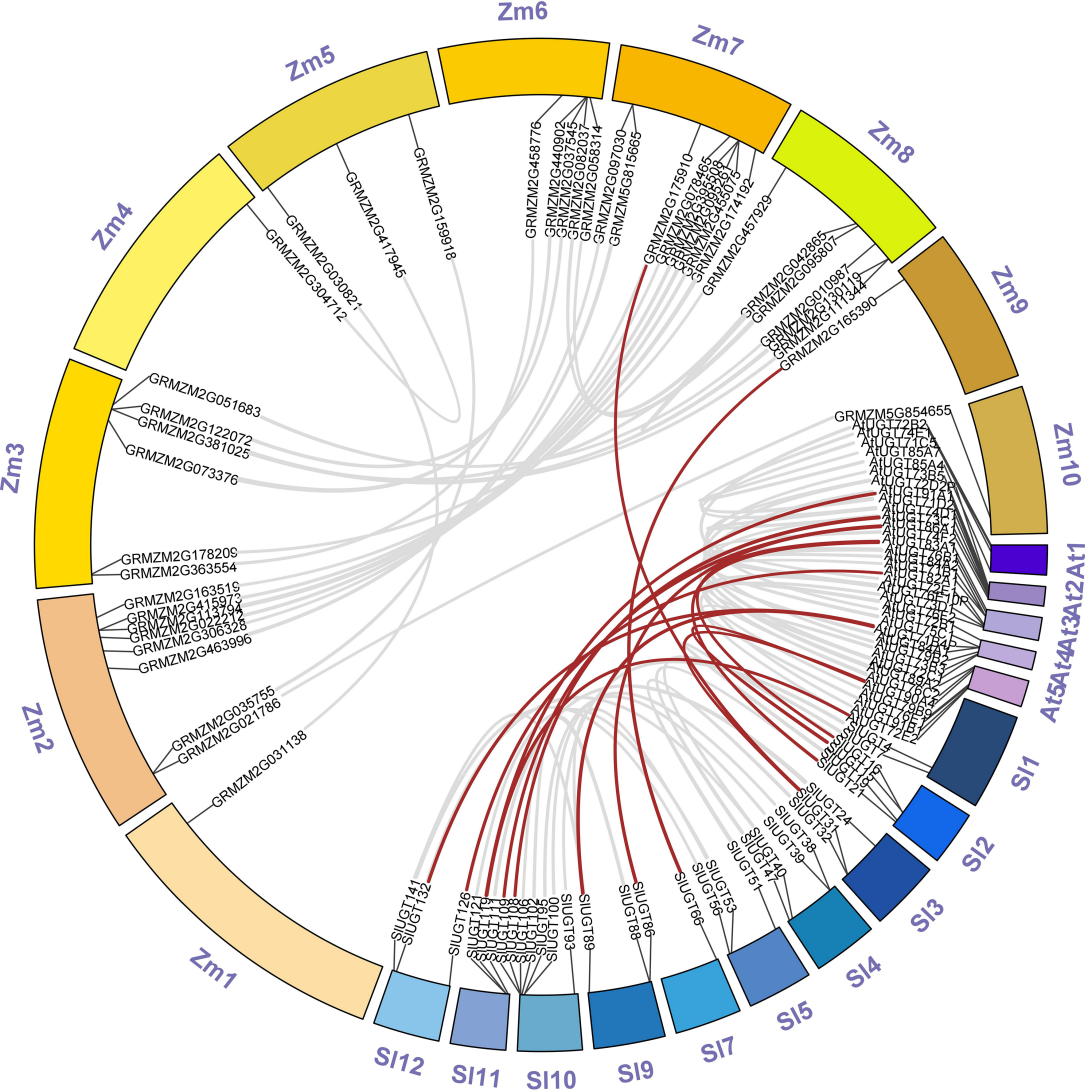
Synteny analysis of UGT genes between tomato and two representative plant species (*Arabidopsis thaliana* and *Z. mays*). “Sl” was on behalf of chromosomes of *Solanum lycopersicum*, “At” was on behalf of chromosomes of *A. thaliana* and “Zm” was on behalf of chromosomes of *Z. mays*.

Multiple sequence alignment revealed that SlUGT proteins were highly conserved. The phylogenetic tree of UGT genes in the tomato, *Z. mays*, and *A. thaliana* was constructed by the neighbor-joining (NJ) method (One UGT gene was selected from each subfamily of *Z. mays*, and *A. thaliana*). The results indicated a classification of 16 major groups (A–P) in tomatoes, including two newly discovered groups (O and P), which were absent in *Arabidopsis* but present in higher plants like maize (Figure 5). These UGT groups (A–P) consisted 26, 2, 2, 18, 18, 2, 11, 5, 2, 1, 5, 18, 3, 1, 25, and 4 members, respectively. To clarify the evolutionary relationship of the SlUGT gene family, we constructed a collinear map between the tomato and one dicotyledon (*A. thaliana*) and one monocotyledon (maize)—12 collinear relationships were found between the UGT of tomato and dicotyledons and only two with monocotyledons (Figure 6).

### cis-Elements in the Promoter Regions of SlUGT Genes

**Figure 7:**
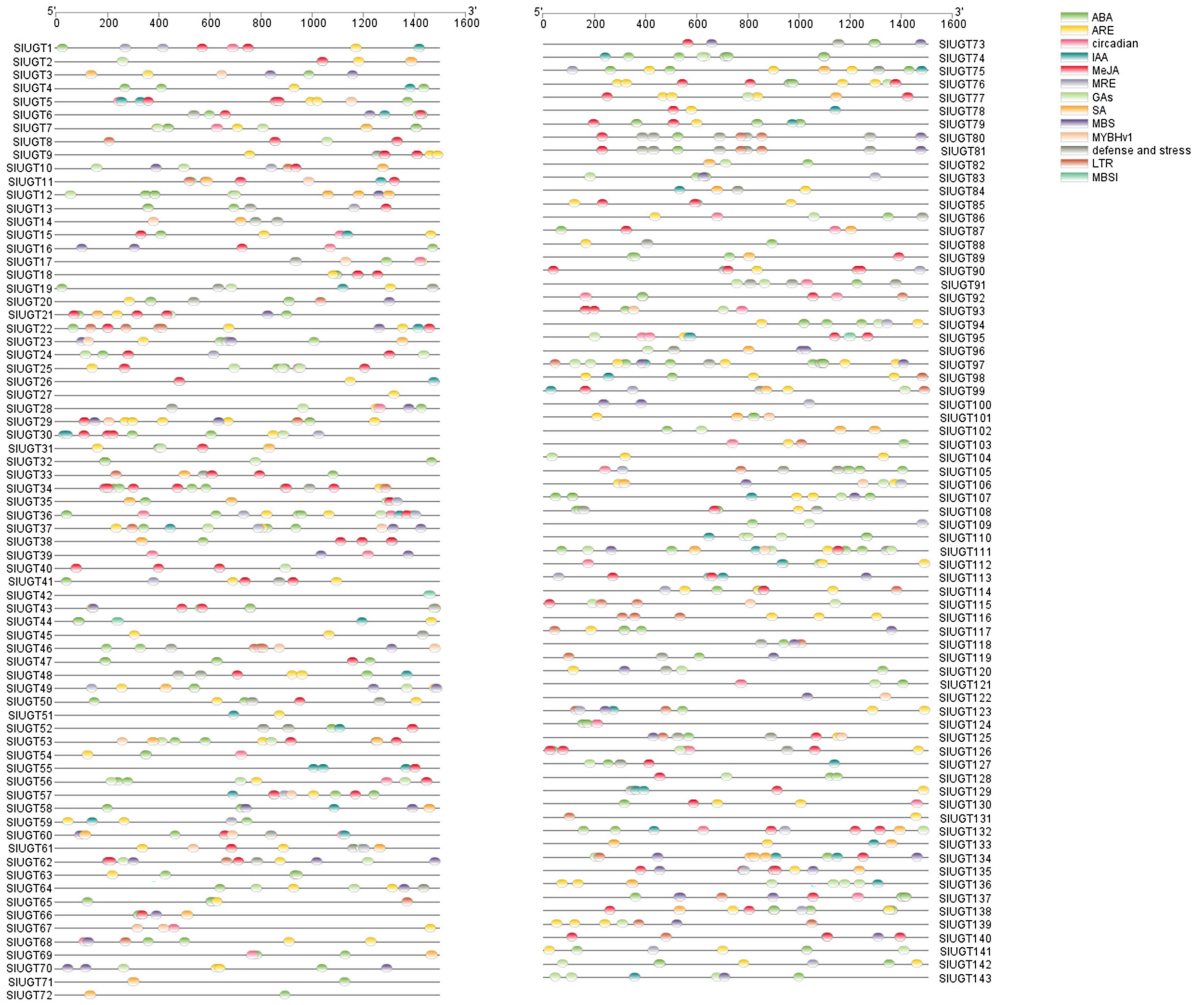
Analysis of the cis-acting elements in SlUGT genes. Different colors represented different cis-acting elements and their position in the SlUGT genes.

To study the regulatory mechanism of the SlUGT gene, the sequence of the promoter codon 2000 bp upstream of (ATG) was scanned in the PlantCARE database, and a variety of cis-acting elements related to plant hormones and stress responses were obtained (Figure 7). Hormone response elements were mainly induced by Abscisic Acid (ABA), methyl jasmonate (MeJA), gibberellin (GA), indole acetic acid (IAA), and salicylic acid (SA). Among them, 108 SlUGT genes with ABA response elements were found, accounting for 75.5% of the total genes. In addition, 72 SlUGT genes containing MeJA response elements and 63 SlUGT genes containing GA elements were revealed. Furthermore, 47 SlUGT genes including IAA response elements and 40 SlUGT genes with at least one SA response element were discovered. Interestingly, the predicted stress response elements included MBS (drought induction), DRE (drought induction), LTR (low-temperature response), ARE (anaerobic induction), and defense and stress-responsive elements, among which, SlUGT genes containing ARE elements were the most frequent. Additionally, a large number of light response-regulatory elements (such as MRE), circadian response elements, and MYB (drought induction) binding sites (such as MBSI) were revealed in the promoter regions of several SlUGT genes. These results demonstrated that SlUGT genes were involved in response to a variety of stresses and hormones, to adapt to diverse environmental changes.

### Gene Structure, Conserved Domain, and Motif Pattern of SlUGT Genes

**Figure 8:**
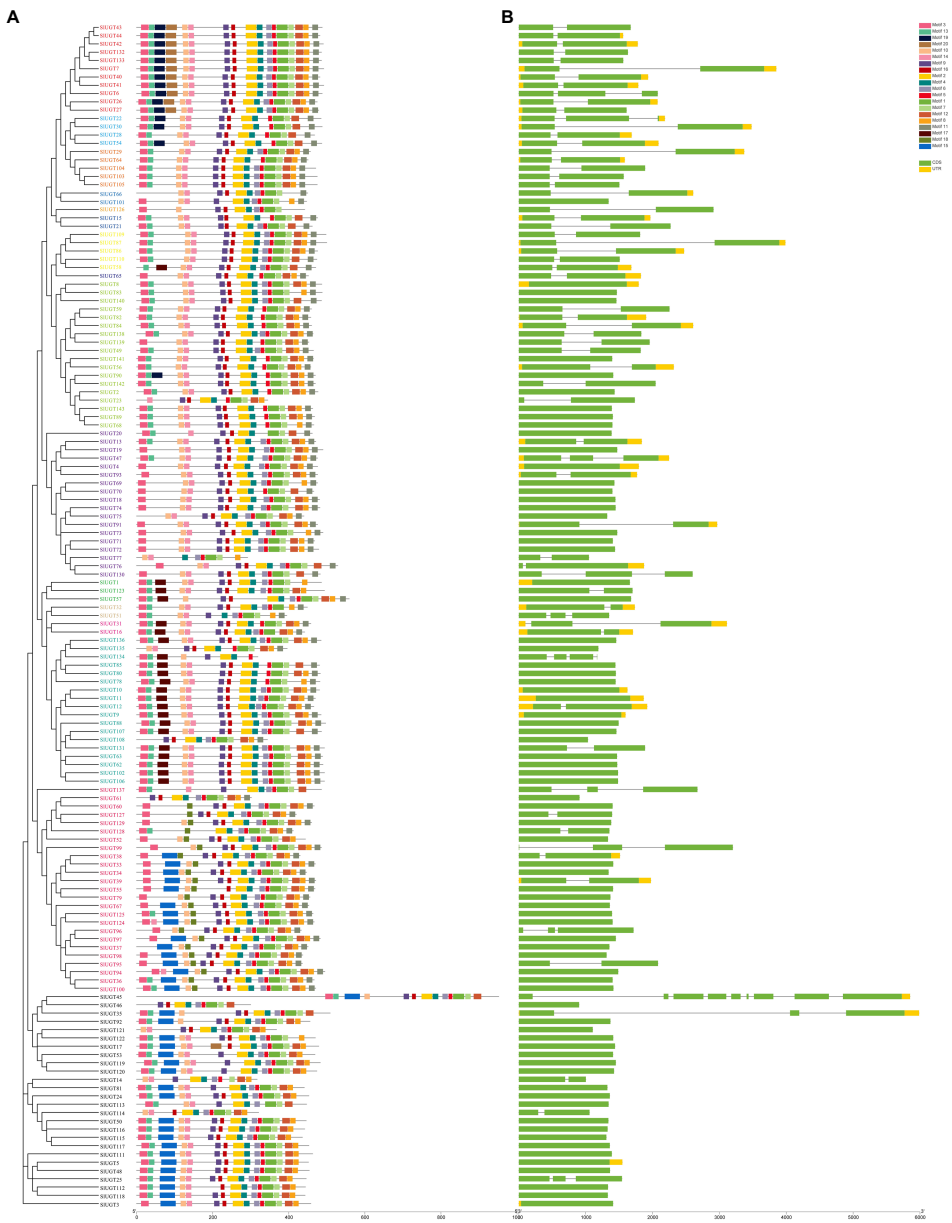
Gene structure and architecture of conserved motifs in SlUGT genes. **(A)** The motif composition of SlUGT proteins. Different colors represented different motifs. **(B)** Exon-intron structure and conserved domain of SlUGT genes.

To identify the conserved structure of the SlUGT protein, 20 motifs were predicted by MEME motif analysis. The results revealed that most SlUGT proteins contained 11–17 motifs, while *SlUGT14*, *SlUGT23*, and *SlUGT126* contained 10 motifs, *SlUGT46* contained nine motifs, *SlUGT77* and *SlUGT134* contained only eight motifs (Figure 8). To understand the evolution of the SlUGT family, we visualized the exon/intron structure of the SlUGT gene (Figure 8). Most UGT genes contained 1–3 exons., and only *UGT45* contained nine exons. Therefore, the gene structure and conserved motif analyses demonstrated that the SlUGTs were highly conserved.

### Expression Patterns of SlUGTs in Different Tissues

**Figure 9:**
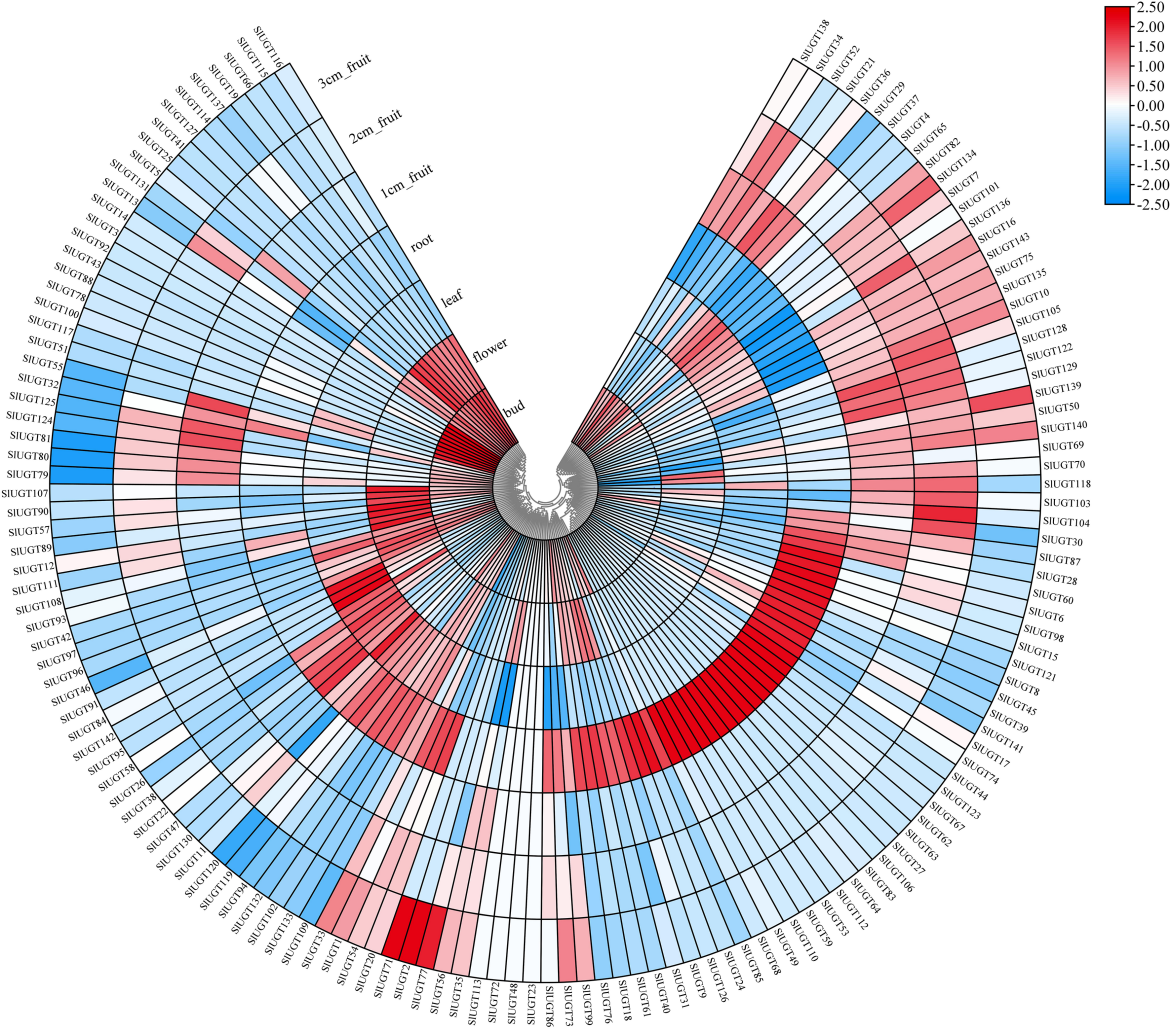
Expression profiles of SlUGT genes in different tissues. The expression levels were based on the transcriptome data. All gene expression levels were log scaled by row.

To study the tissue-specific expression patterns of members of the SlUGT gene family, we analyzed the transcriptome data of UGTs present in roots, leaves, flowers, buds, and fruits in wild tomatoes (*Solanum pimpinellifolium* L.). As shown in Figure 9, most of the SlUGT genes, except for *SlUGT23*, *SlUGT48*, *SlUGT72*, and *SlUGT113*, could be expressed in tissue sites. Among them, 90 UGT genes were expressed in all tissues, accounting for 62.9% of the total UGT genes, while SlUGT3and SlUGT14, which were only expressed in buds, *SlUGT53*, *SlUGT59*, *SlUGT110*, and *SlUGT112*, which were only expressed in roots, and *SlUGT2*, *SlUGT71* were only expressed in fruits. These results suggested that SlUGT was expressed in a tissue-specific manner in the tomato.

### Expression Profile of SlUGT Genes in Transcriptome Data Under Different Treatments

**Figure 10:**
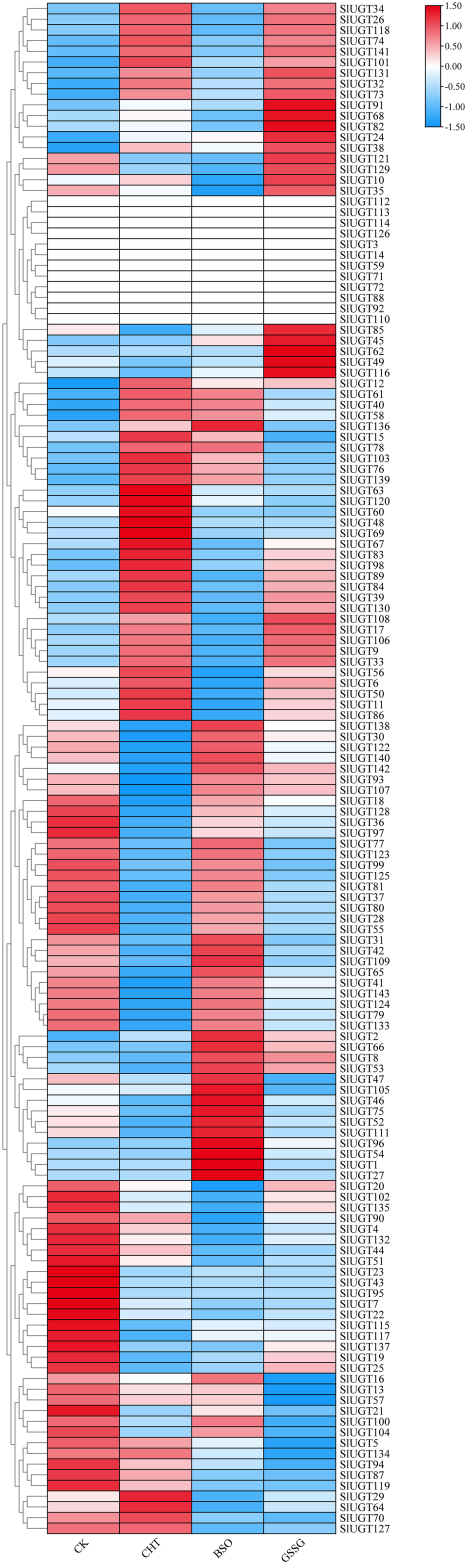
Expression profile of SlUGT genes in transcriptome data under different treatments. All gene expression levels were log scaled by row.

Based on the transcriptome data, we analyzed the SlUGT gene family members’ response to CHT and glutathione. The expression of 52 genes was upregulated, while that of 75 genes was downregulated after CHT treatment compared with the control (Figure 10). The transcription of 59 UGT genes was induced, however, that of 69 UGT genes was decreased by BSO treatment compared with the only CHT treatment. Similarly, the transcription of 67 UGT genes was induced, however, that of 61 UGT genes was decreased by GSSG treatment compared with the only CHT treatment (Figure 10). It showed that UGT genes responded to glutathione under CHT treatment.

### Analysis of the Expression Profile of SlUGT Genes

**Figure 11:**
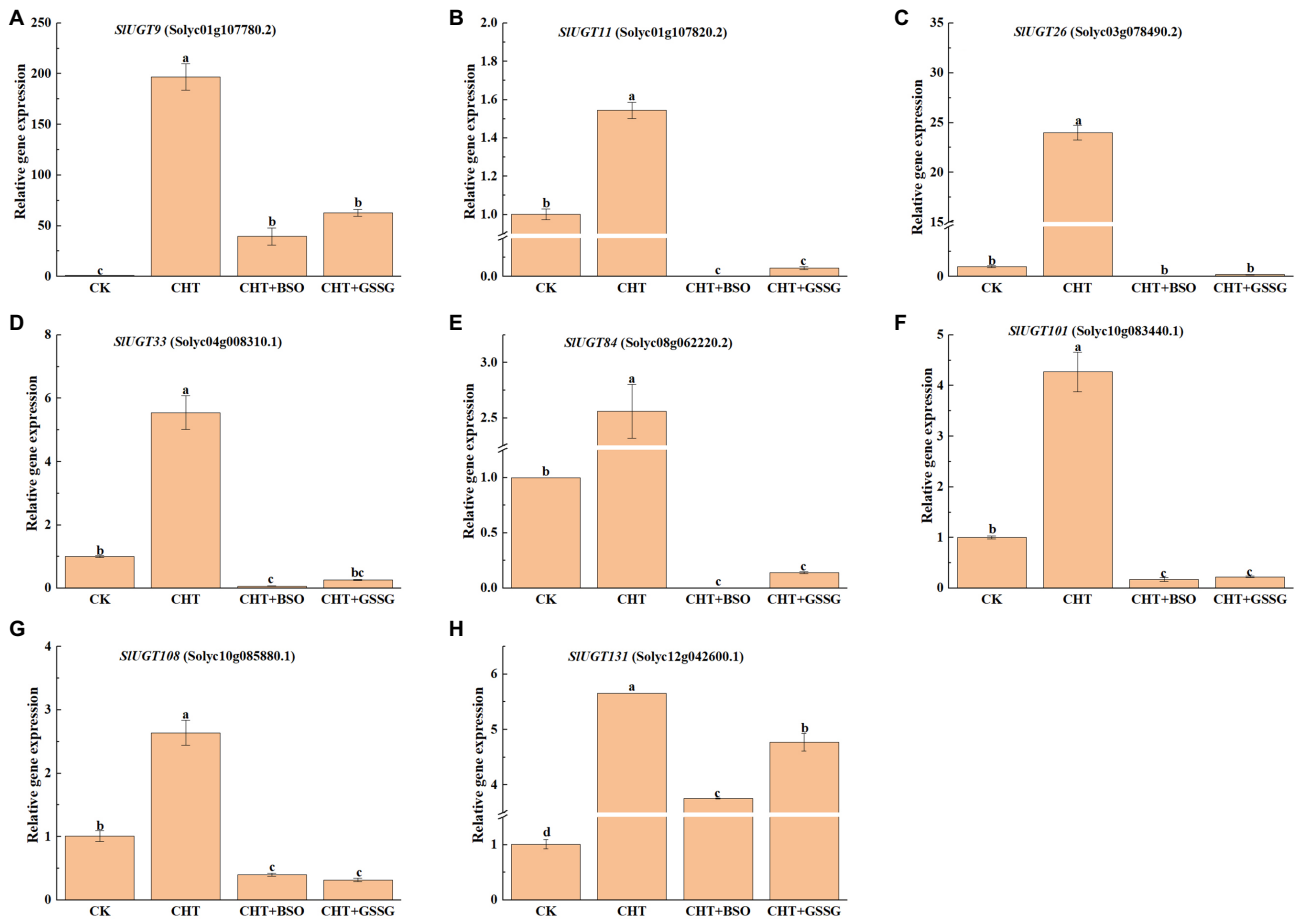
Expression profiles of eight representative SlUGT genes in response to different treatments. **(A)** SlUGT9 expression. **(B)** SlUGT11 expression. **(C)** SlUGT26 expression. **(D)** SlUGT33 expression. **(E)** SlUGT84 expression. **(F)** SlUGT101 expression. **(G)** SlUGT108 expression. **(H)** SlUGT131 expression. Error bars represented the SD (*n* = 3). According to Duncan’s multiple tests, bars with different letters were significantly different (*p* < 0.05).

To confirm the effect of glutathione on SlUGT gene expression under CHT treatment, we selected several representative SlUGT genes and explored their genetic expression after treatment with BSO and GSSG under CHT treatment by qRT-PCR. After CHT treatment, the expression of eight UGT genes selected was significantly up-regulated compared with control, while after BSO and GSSG treatment, the expression of eight UGT genes was significantly inhibited compared with only CHT treatment (Figure 11), suggesting that they might be the key UGT genes induced by glutathione involved in the response to CHT stimulation in tomato.

### Expression of UGT Gene and Its Related Transcription Factors Under Different Treatments

**Figure 12:**
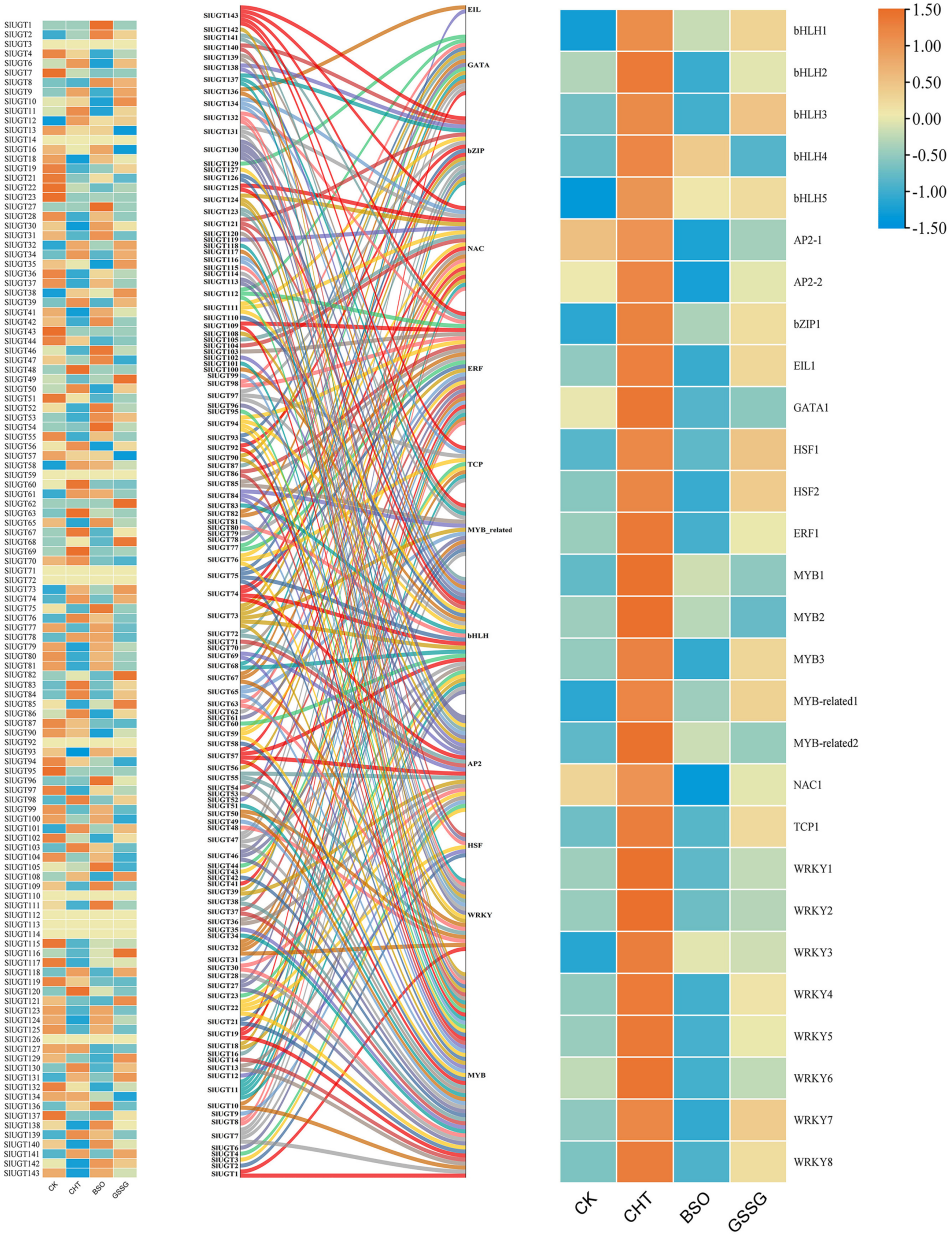
Network analysis of tomato UGT genes and transcription factors and Expression profiles of different SlUGT genes and transcription factors in response to different treatments. Different colors represent different SlUGT genes.

To further explore the regulatory mechanism of the SlUGT gene, we predicted that a total of 12 transcription factor (TF) families interacted with the SlUGT gene (Figure 12). Among all SlUGT genes, nine transcription factors were predicted to interact with *SlUGT73* and *SlUGT75*, with the largest number. While the transcription factor MYB interacted with 51 UGT genes, accounting for 35.7%. Interestingly, transcriptome data showed that each of the 12 transcription factor families contained genes that could be induced by CHT, while inhibited by BSO or GSSG treatment. The above results revealed that the UGT gene could participate in the regulation of plant growth and development and stress response through interaction with transcription factors in tomatoes, which meant that the SlUGT gene played an important role in plant detoxification through interaction with transcription factors.

## Discussion

Fungicides are an essential part of intensive crop production, especially for economically important crop varieties (Alengebawy et al., 2021). It has been shown that approximately 50,000 plant pathogens can contribute to significant crop losses (Zhang, 2018). CHT is a widely used fungicide with an astonishingly high annual use (about 8,000 tons/year, such as in China; Wang et al., 2022). However, the massive use of pesticides will not only produce benefits but also cause excessive pesticide residues, which not only endangers the growth of plants and human health but also has a serious impact on the whole ecological environment (Ramel et al., 2012). It has been found that melatonin could promote BPA metabolism by improving glutathione-dependent detoxification of tomatoes (Kanwar et al., 2020). As a key antioxidant and redox buffer, GSH is widely distributed in all the cell compartments in plant (Noctor et al., 2012). In this study, we utilized BSO, a kind of glutathione inhibitor, and GSSG, Oxidized glutathione, to explore the effect of glutathione on the tolerance of CHT phytotoxicity in tomatoes. We found that BSO reduced NPT content under CHT stress, and improved CHT accumulation in tomatoes, suggesting that glutathione promoted CHT metabolism in tomato plants. Similarly, GSSG treatment could also increase the accumulation of chlorothalonil, although there was no significant difference in NPT content between GSSG treatment and pesticide treatment alone. Jung et al. (2021) also found that foliar application of glutathione could reduce cadmium-induced oxidative stress.

The production of ROS production (H_2_O_2_ and O_2_^−^) in plant cells increased under stress. Studies have shown that glutathione can improve the salt tolerance of sorghum by scavenging ROS (Punia et al., 2021). In addition, a study on upland cotton plants under lead (Pb) stress proved the importance of GSH in HMS detoxification (Khan et al., 2016). Our results also show that exposure to the CHT leads to an increase of reactive oxygen species in tomatoes, suggesting that the CHT induces oxidative stress in tomatoes through the excessive accumulation of ROS. Consistent with the ROS content, an increased CHT accumulation increased the antioxidant enzymes activity and the content of non-enzymatic substances, indicating that ROS levels may exceed antioxidant elimination capacity, although plants can improve their scavenging capacity of ROS to resist again stress. Interestingly, when glutathione was inhibited, the content of ROS in tomatoes increased further, while the content of non-enzymatic antioxidants and the activity of antioxidant enzymes decreased significantly, revealing that glutathione enhanced antioxidant capacity to alleviate the CHT-induced oxidative stress in tomatoes, and then degraded the CHT residue in tomato.

In general, xenometabolism in both animals and higher plants consisted of three processes: transformation (phase I), binding (phase II), and compartmentalization (phase III; Yu et al., 2013). Glutathione (GSH) is a kind of low-molecular-weight thiol, which can be used as the substrate of GST to participate in phase II detoxification of plants (Geu-Flores et al., 2011). It has been found that ABC transporters can participate in the detoxification of CHT in tomatoes (Szabados and Savour’e, 2010). In addition, Hou et al. (2018) observed that the CHT degradation promoted by EBR was related to the increase of GST activity. In the present study, the expression of detoxification genes (including P450, GST, UGT, and ABC) increased after CHT treatment compared with the control. However, BSO and GSSG administration substantially inhibited the expression of all these genes compared with the only CHT treatment. It revealed that glutathione could degrade the residual CHT in tomatoes by inducing the expression of detoxification genes related to glutathione.

It is reported that UGT also plays a role in various abiotic stress and xenobiotic detoxification in plants (Mamoon Rehman et al., 2016). However, the role of UGT genes in tomatoes has not been studied. Previous studies have identified a total of 145 UGT genes in *C. grandis*,147 UGT genes in *Z. mays*, 179 UGT genes in *Triticum aestivum*, and 180 UGT genes in *Oryza sativa* (Caputi et al., 2012; Li et al., 2014; He et al., 2018; Wu et al., 2020). In the present study, a total of 143 UGT genes were identified in tomato, these UGT genes were divided into 16 groups, including 14 highly conserved groups (A–N) and two newly discovered groups O and P, which were absent in Arabidopsis, while Q Group only existed in maize, suggesting that this group might exist only in monocotyledons and could play a significant role in the UGT gene evolution of monocotyledons. In addition, Arabidopsis and tomato belong to dicotyledonous plants. There are a large number of collinear relationships between them, while there are only two collinear relationships with the monocotyledonous plant (maize). This result is consistent with the evolutionary relationship between dicotyledonous and monocotyledonous plants.

Introns are important components of genes. Although they are not involved in protein coding, the acquisition or loss of introns and the insertion position of introns relative to protein sequences are generally considered to be key clues to understanding the evolutionary diversity of gene families (Rogozin et al., 2000). Gene structure and motif analysis showed that the SlUGT protein was highly conserved, and most members of the SlUGT gene family contained a typical motif, and motif4 existed in all SlUGT genes. SlUGT genes of the same clade contained a similar number of exons/introns and motifs. In addition, some clades included a unique motif, such as, motif 20 was specific to group G, which might be the reason that these clades could perform specific functions.

Abiotic stress resistance is one of the important traits during tomato breed improvement (Cortina et al., 2005; Almaghamsi et al., 2021). In recent years, it has been found that UGT genes of *Melilotus albus* may participate in various defense responses against biotic and abiotic stresses (Duan et al., 2021). It has also been found that UGT genes could regulate tolerance under FHB (Fusarium head blight) in wheat (He et al., 2018). Here, we also found several plant hormone and stress response-regulatory elements in the promoter region of SlUGT genes. It showed that UGT genes in tomatoes were related to plant hormones and stress regulation. At the same time, transcriptome data revealed that most UGT genes could be induced or inhibited by CHT, and the expression of these genes was also changed after pretreatment with BSO or GSSG. This suggested that the SlUGT gene family played an important role in plant detoxification, which might be regulated by glutathione. QRT-PCR analysis further confirmed the above conclusions. Interestingly, most of the UGT genes induced by CHT belonged to subgroup D, indicating that this subgroup played a major role in tomato detoxification.

Transcription factors (TFs) are important proteins, which bind to specific DNA motifs, regulate gene expression and play an important role in plant stress response (Wu et al., 2022). The present study found that 121 tomato UGT genes interacted with 12 different transcription factors, 90% of which were regulated by glutathione under CHT stress. It could be seen that glutathione might promote the metabolism of pesticides in tomatoes by regulating the interaction between the UGT gene and transcription factors. In addition, among them, 54 UGT genes had complex relationship networks with MYB transcription factors, and the MYB transcription factor has been proved to be related to abiotic stress in plants (Dar et al., 2022). It has been reported that the UGT gene could work with transcription factors to affect the stress response of cassia seeds (Deng et al., 2018). Therefore, SlUGT genes could participate in the regulation of stress through the interaction with these transcription factors.

## Conclusion

In summary, we found that CHT treatment-induced oxidative stress in tomatoes by producing ROS. However, glutathione increased the activity of antioxidative enzymes, the content of non-enzymatic substances, and the expression of detoxification genes to scavenge ROS and reduce the accumulation of CHT in tomato plants. In addition, we presented the first instance of comprehensive information about UGT family genes in tomatoes. The phylogenetic analysis showed that 143 SlUGT genes could be grouped into 16 subfamilies (Group A–P) with conserved domains and motifs. Most of the identified genes are expressed in different tissue types. The promoter region of SlUGT genes contained several plant hormone- and stress response-regulatory elements. Transcriptome data and qRT-PCR results concurred that most SlUGT genes could be induced by CHT, and the expression of these genes is regulated by glutathione. We also found SlUGT gene could participate in plant detoxification through interaction with transcription factors, which conferred a new direction for studies on the function of the SlUGT gene.

## Data Availability Statement

The data presented in the study are deposited in the NCBI repository, available at: https://dataview.ncbi.nlm.nih.gov/object/PRJNA835910?reviewer=kt60ukoga0butbtus3p7lk4nt.

## Author Contributions

GY: conceptualization, writing-review and editing, and funding acquisition. QC: methodology, formal analysis, writing-original draft. FC: investigation. HL: data curation. JL: software. RC: investigation. CR: data curation. JW: writing-review and editing. YZ: resources and funding acquisition. FY: resources. YS: resources. All authors have read and agreed to the published version of the manuscript.

## Funding

This work was financially supported by the Postdoctoral Scientific Research Developmental Fund of Heilongjiang (LBH-Q20052), the Fund Program for Overseas Returnees, National Natural Science Foundation of China (31301769), and Heilongjiang Bayi Agricultural University PhD Research Initiation Fund, Heilongjiang Bayi Agricultural University Support Program for San Heng San Zong (TDJH202004), the Natural Science Foundation of Heilongjiang Province (QC2018023).

## Conflict of Interest

The authors declare that the research was conducted in the absence of any commercial or financial relationships that could be construed as a potential conflict of interest.

## Publisher’s Note

All claims expressed in this article are solely those of the authors and do not necessarily represent those of their affiliated organizations, or those of the publisher, the editors and the reviewers. Any product that may be evaluated in this article, or claim that may be made by its manufacturer, is not guaranteed or endorsed by the publisher.

## Footnotes

^1^ http://www.arabidopsis.org/

^2^ http://smart.embl-heidelberg.de/

^3^ http://www.ncbi.nlm.nih.gov/protein/

^4^ http://phylogeny.lirmm.fr/phylo_cgi/one_task.cgi?task_type=gblocks

^5^ http://gsds.cbi.pku.edu.cn/

^6^ http://meme-suite.org/tools/meme

^7^ http://plantregmap.gao-lab.org/index-chinese.php
