# Supplementary material for: Glutathione Promotes Degradation and Metabolism of Residual Fungicides by Inducing UDP-Glycosyltransferase Genes in Tomato
Source: Front Plant Sci. 2022 Jul 1;13:893508. doi: 10.3389/fpls.2022.893508 (PMC9289782; doi:10.3389/fpls.2022.893508)
Supplement: Supplementary file 1 [file Data_Sheet_1.docx]

**Table S1**

**Physicochemical parameters of *SlUGT* genes**

| Gene Name | Gene ID | Chromosomes | Number of aa | Formula | Kda Molecular weight | Theoretical pl | Grand average of hydropathicity (GRAVY) | Aliphatic index | Istability inndex | Subcellular localization |
| --- | --- | --- | --- | --- | --- | --- | --- | --- | --- | --- |
| SlUGT1 | Solyc01g006670.2 | 1 | 486 | C_2503_H_3834_N_636_O_715_S_22_ | 54981.13 | 5.04 | -0.076 | 91.23 | 41.34 | nucl, cyto, cysk |
| SlUGT2 | Solyc01g066100.1 | 1 | 477 | C_2439_H_3785_N_623_O_706_S_21_ | 53804.89 | 6.12 | -0.092 | 92.12 | 36.9 | nucl, cyto, golg |
| SlUGT3 | Solyc01g067350.2 | 1 | 458 | C_2338_H_3667_N_599_O_687_S_14_ | 51608.28 | 5.68 | -0.211 | 95.11 | 39.61 | cyto, chlo, nucl |
| SlUGT4 | Solyc01g095620.2 | 1 | 476 | C_2358_H_3736_N_624_O_691_S_19_ | 52492.51 | 5.63 | -0.073 | 98.3 | 41.36 | chlo, cyto, nucl |
| SlUGT5 | Solyc01g095760.2 | 1 | 452 | C_2330_H_3654_N_598_O_668_S_23_ | 51469.63 | 5.91 | -0.205 | 91 | 46.91 | pero, chlo, nucl |
| SlUGT6 | Solyc01g105350.1 | 1 | 488 | C_2476_H_3898_N_650_O_723_S_28_ | 55237.79 | 6.04 | -0.137 | 92.66 | 39.19 | cyto, plas, chlo |
| SlUGT7 | Solyc01g105360.2 | 1 | 492 | C_2468_H_3832_N_654_O_716_S_29_ | 55051.27 | 5.61 | -0.083 | 91.36 | 35.13 | pero, chlo, nucl |
| SlUGT8 | Solyc01g107560.2 | 1 | 487 | C_2444_H_3808_N_628_O_709_S_22_ | 54038.22 | 5.83 | -0.134 | 89.49 | 38.26 | chlo, nucl, cyto |
| SlUGT9 | Solyc01g107780.2 | 1 | 485 | C_2410_H_3795_N_645_O_721_S_29_ | 54271.27 | 5.47 | -0.245 | 81.79 | 34.42 | chlo, nucl, cyto |
| SlUGT10 | Solyc01g107810.2 | 1 | 481 | C_2456_H_3806_N_638_O_724_S_20_ | 54496.28 | 5.22 | -0.208 | 84.91 | 42.55 | chlo, cyto, nucl |
| SlUGT11 | Solyc01g107820.2 | 1 | 470 | C_2359_H_3677_N_631_O_705_S_25_ | 52959.45 | 5.38 | -0.291 | 77.43 | 41.07 | chlo, cyto, extr |
| SlUGT12 | Solyc01g107830.2 | 1 | 466 | C_2397_H_3721_N_623_O_684_S_24_ | 52980.12 | 5.71 | -0.162 | 87.47 | 47.32 | cyto, chlo, cysk |
| SlUGT13 | Solyc02g063000.2 | 2 | 471 | C_2365_H_3782_N_628_O_699_S_21_ | 52871.09 | 5.8 | -0.134 | 99.53 | 43.64 | nucl, cyto, chlo |
| SlUGT14 | Solyc02g065670.1 | 2 | 317 | C_1643_H_2580_N_430_O_473_S_13_ | 36341.94 | 5.28 | -0.17 | 97.98 | 48.19 | cyto, cysk, chlo |
| SlUGT15 | Solyc02g066960.2 | 2 | 476 | C_2387_H_3758_N_616_O_699_S_23_ | 53007.18 | 6.03 | -0.056 | 95.86 | 33.97 | chlo, extr, nucl |
| SlUGT16 | Solyc02g067690.2 | 2 | 442 | C_2251_H_3516_N_578_O_647_S_19_ | 49637.3 | 5.51 | -0.028 | 92.13 | 43.34 | chlo, vacu, extr |
| SlUGT17 | Solyc02g070020.1 | 2 | 479 | C_2436_H_3751_N_645_O_715_S_17_ | 54058.49 | 5.28 | -0.204 | 89.08 | 42.42 | cyto, E.R._plas, plas |
| SlUGT18 | Solyc02g081690.1 | 2 | 481 | C _2446_ H _3781_ N _619_ O _701_ S _18_ | 53652.73 | 5.66 | -0.006 | 96.44 | 45.54 | chlo, cyto, nucl |
| SlUGT19 | Solyc02g085660.1 | 2 | 490 | C_2427_H_3884_N_638_O_731_S_23_ | 54434.75 | 5.5 | -0.114 | 98.59 | 40.75 | chlo, nucl, cyto |
| SlUGT20 | Solyc02g088500.1 | 2 | 462 | C_2310_H_3608_N_594_O_681_S_21_ | 51270.89 | 4.87 | -0.019 | 97.49 | 37.42 | cyto, chlo, nucl |
| SlUGT21 | Solyc02g088750.1 | 2 | 462 | C_2334_H_3618_N_604_O_672_S_21_ | 51565.3 | 5.47 | -0.083 | 92.01 | 38.28 | chlo, cyto, extr |
| SlUGT22 | Solyc02g091350.2 | 2 | 485 | C_2489_H_3873_N_657_O_726_S_27_ | 55482.72 | 5.43 | -0.163 | 93.2 | 45.7 | chlo, vacu, nucl |
| SlUGT23 | Solyc03g058370.1 | 3 | 345 | C_1759_H_2747_N_461_O_499_S_26_ | 39170.51 | 6.97 | -0.057 | 88.41 | 44.36 | mito, chlo_mito, cyto_mito |
| SlUGT24 | Solyc03g071850.1 | 3 | 453 | C_2317_H_3656_N_608_O_678_S_19_ | 51487.32 | 5.57 | -0.276 | 92.67 | 47.73 | chlo, cyto, nucl |
| SlUGT25 | Solyc03g078240.1 | 3 | 446 | C_2270_H_3567_N_615_O_657_S_18_ | 50563.1 | 6.16 | -0.229 | 94.6 | 46.17 | cyto, chlo, chlo_mito |
| SlUGT26 | Solyc03g078490.2 | 3 | 475 | C_2433_H_3758_N_640_O_698_S_26_ | 53976.03 | 5.7 | -0.231 | 84.15 | 42.97 | chlo, nucl, cyto |
| SlUGT27 | Solyc03g078500.2 | 3 | 479 | C_2445_H_3782_N_638_O_701_S_28_ | 54228.46 | 6.03 | -0.147 | 84.43 | 35.67 | pero, chlo, mito |
| SlUGT28 | Solyc03g078770.2 | 3 | 468 | C_2359_H_3705_N_627_O_695_S_24_ | 52739.59 | 5.54 | -0.149 | 90.17 | 39.1 | nucl, cyto, chlo |
| SlUGT29 | Solyc03g078810.2 | 3 | 457 | C_2337_H_3644_N_604_O_660_S_17_ | 51307.31 | 5.81 | 0.065 | 101.55 | 45.85 | cyto, E.R., nucl |
| SlUGT30 | Solyc03g083650.2 | 3 | 488 | C_2488_H_3884_N_636_O_723_S_24_ | 55043.48 | 5.41 | -0.061 | 96.62 | 40.24 | chlo, chlo_mito |
| SlUGT31 | Solyc03g114180.2 | 3 | 458 | C_2353_H_3641_N_611_O_658_S_28_ | 51915.17 | 6.21 | -0.099 | 83.28 | 43.91 | chlo, E.R., nucl |
| SlUGT32 | Solyc03g114710.2 | 3 | 449 | C_2279_H_3528_N_606_O_641_S_14_ | 50121.6 | 6.14 | -0.072 | 96.41 | 43.91 | chlo, cyto |
| SlUGT33 | Solyc04g008310.1 | 4 | 470 | C_2341_H_3706_N_640_O_707_S_23_ | 52866.42 | 5.19 | -0.19 | 92.49 | 58.27 | nucl, cyto, plas |
| SlUGT34 | Solyc04g008330.1 | 4 | 447 | C_2260_H_3578_N_618_O_673_S_19_ | 50784.15 | 5.83 | -0.222 | 96.33 | 49.01 | chlo, nucl, cysk_nucl |
| SlUGT35 | Solyc04g010110.2 | 4 | 509 | C_2586_H_4068_N_644_O_732_S_25_ | 56694.12 | 6.62 | 0.02 | 96.72 | 37.64 | cyto, E.R., nucl |
| SlUGT36 | Solyc04g016190.1 | 4 | 467 | C_2396_H_3727_N_645_O_680_S_21_ | 53122.12 | 7.74 | -0.214 | 86.77 | 46.08 | chlo, nucl |
| SlUGT37 | Solyc04g016200.1 | 4 | 451 | C_2308_H_3554_N_630_O_668_S_24_ | 51584.87 | 6.35 | -0.351 | 79.31 | 51.66 | chlo, nucl, mito |
| SlUGT38 | Solyc04g016210.2 | 4 | 430 | C_2151_H_3403_N_587_O_633_S_20_ | 48256.43 | 6.05 | -0.2 | 91.4 | 58.42 | nucl, cyto, chlo |
| SlUGT39 | Solyc04g016230.2 | 4 | 471 | C_2362_H_3703_N_645_O_697_S_25_ | 53089.79 | 5.81 | -0.205 | 88.17 | 60.85 | cyto, E.R., nucl |
| SlUGT40 | Solyc04g074330.2 | 4 | 482 | C_2464_H_3818_N_640_O_715_S_27_ | 54712.9 | 5.46 | -0.142 | 89.54 | 43.68 | chlo, nucl, cyto |
| SlUGT41 | Solyc04g074340.2 | 4 | 491 | C_2476_H_3868_N_640_O_715_S_27_ | 54907.43 | 5.95 | -0.149 | 90.37 | 35.84 | cyto, nucl, cysk |
| SlUGT42 | Solyc04g074340.2 | 4 | 492 | C_2481_H_3855_N_647_O_727_S_23_ | 55116.18 | 5.36 | -0.053 | 95.28 | 42.57 | chlo, vacu, nucl |
| SlUGT43 | Solyc04g074360.1 | 4 | 488 | C_2458_H_3865_N_637_O_726_S_28_ | 54854.24 | 5.67 | -0.21 | 87.11 | 40.78 | cyto, chlo, nucl |
| SlUGT44 | Solyc04g074390.2 | 4 | 482 | C_2440_H_3769_N_627_O_711_S_25_ | 54065.04 | 5.46 | -0.222 | 84.56 | 38.13 | chlo, cyto, nucl |
| SlUGT45 | Solyc04g079030.2 | 4 | 952 | C_4997_H_7727_N_1271_O_1407_S_48_ | 109659.87 | 6.14 | -0.16 | 91.31 | 40.52 | cyto, plas, vacu |
| SlUGT46 | Solyc04g079050.1 | 4 | 300 | C_1535_H_2415_N_389_O_438_S_19_ | 33936.54 | 5.58 | -0.075 | 94.77 | 37.4 | cyto, nucl, extr |
| SlUGT47 | Solyc04g080010.2 | 4 | 476 | C_2415_H_3758_N_618_O_705_S_15_ | 53211.02 | 5 | -0.047 | 95.44 | 43.43 | chlo, nucl, cyto |
| SlUGT48 | Solyc04g081830.1 | 4 | 454 | C_2354_H_3717_N_625_O_671_S_18_ | 52087.27 | 8.37 | -0.307 | 90.73 | 48.19 | cyto, nucl, chlo |
| SlUGT49 | Solyc04g082860.1 | 4 | 465 | C_2345_H_3629_N_615_O_688_S_20_ | 52086.52 | 6.13 | -0.157 | 86.6 | 39.8 | nucl, cyto, cysk |
| SlUGT50 | Solyc05g005930.1 | 5 | 446 | C_2288_H_3603_N_585_O_665_S_18_ | 50523.38 | 5.2 | -0.138 | 100.31 | 40.4 | chlo, chlo_mito, cyto |
| SlUGT51 | Solyc05g010710.1 | 5 | 395 | C_1985_H_3131_N_525_O_556_S_11_ | 43599.54 | 6.77 | 0.023 | 100.89 | 38.22 | vacu, chlo, cyto |
| SlUGT52 | Solyc05g012670.1 | 5 | 444 | C_2252_H_3526_N_622_O_674_S_22_ | 50803.85 | 5.87 | -0.359 | 86.22 | 51.99 | chlo, nucl, vacu |
| SlUGT53 | Solyc05g051360.1 | 5 | 469 | C_2446_H_3785_N_639_O_680_S_18_ | 53600.91 | 6.1 | -0.163 | 95.35 | 34.8 | chlo, cyto, vacu |
| SlUGT54 | Solyc05g052870.2 | 5 | 487 | C_2489_H_3881_N_657_O_729_S_28_ | 55570.84 | 5.3 | -0.217 | 89.59 | 45.62 | cyto, E.R., plas |
| SlUGT55 | Solyc05g053120.1 | 5 | 469 | C_2391_H_3713_N_625_O_699_S_26_ | 53232.11 | 5.46 | -0.185 | 89.79 | 43.82 | cyto, nucl, mito |
| SlUGT56 | Solyc05g053820.2 | 5 | 458 | C_2319_H_3634_N_608_O_680_S_19_ | 51521.17 | 5.72 | -0.144 | 95.09 | 41.19 | chlo, nucl, cyto |
| SlUGT57 | Solyc05g053890.1 | 5 | 559 | C_2790_H_4394_N_728_O_834_S_29_ | 62409.7 | 5.88 | -0.196 | 86.14 | 48.44 | chlo, nucl, cyto |
| SlUGT58 | Solyc05g055840.2 | 5 | 471 | C_2404_H_3719_N_641_O_711_S_19_ | 53585.98 | 5.88 | -0.24 | 87.54 | 39.9 | chlo, nucl, mito |
| SlUGT59 | Solyc06g007650.1 | 6 | 460 | C_2346_H_3683_N_607_O_687_S_30_ | 52345.5 | 5.41 | -0.172 | 93.15 | 46.57 | chlo, cyto, nucl |
| SlUGT60 | Solyc06g062290.1 | 6 | 467 | C_2349_H_3704_N_652_O_701_S_22_ | 53000.52 | 5.93 | -0.236 | 93.21 | 47.92 | cyto, nucl, cysk |
| SlUGT61 | Solyc06g062330.1 | 6 | 302 | C_1508_H_2357_N_407_O_459_S_16_ | 34045.72 | 5.32 | -0.363 | 78.05 | 46.74 | nucl, cyto, chlo |
| SlUGT62 | Solyc06g072870.1 | 6 | 490 | C_2460_H_3950_N_666_O_708_S_24_ | 54953.9 | 6.08 | -0.097 | 100.27 | 40.12 | nucl, chlo, cysk_nucl |
| SlUGT63 | Solyc06g072880.1 | 6 | 489 | C_2471_H_3914_N_660_O_716_S_22_ | 55029.57 | 5.36 | -0.074 | 98.9 | 40.85 | chlo, nucl, cysk_nucl |
| SlUGT64 | Solyc06g076550.2 | 6 | 451 | C_2323_H_3619_N_601_O_669_S_21_ | 51344.17 | 5.62 | -0.07 | 97.67 | 42.3 | nucl, cyto, chlo |
| SlUGT65 | Solyc06g082300.2 | 6 | 452 | C_2343_H_3594_N_590_O_673_S_22_ | 51501.18 | 5.38 | -0.062 | 90.53 | 42.32 | nucl, cysk, chlo |
| SlUGT66 | Solyc07g006720.2 | 7 | 450 | C_2239_H_3456_N_574_O_651_S_18_ | 49408.61 | 5.82 | -0.015 | 88.76 | 32.07 | chlo, cyto, vacu |
| SlUGT67 | Solyc07g006800.1 | 7 | 453 | C_2308_H_3550_N_612_O_674_S_23_ | 51392.65 | 6.16 | -0.269 | 78.3 | 55.38 | chlo, nucl, plas |
| SlUGT68 | Solyc07g008230.1 | 7 | 466 | C_2382_H_3685_N_613_O_693_S_24_ | 52767.59 | 5.98 | -0.165 | 88.84 | 33.54 | chlo, extr, vacu |
| SlUGT69 | Solyc07g043050.1 | 7 | 476 | C_2445_H_3758_N_626_O_702_S_17_ | 53699.53 | 4.96 | -0.011 | 95.06 | 47.58 | E.R., cyto, E.R._plas |
| SlUGT70 | Solyc07g043060.1 | 7 | 466 | C_2383_H_3685_N_617_O_693_S_15_ | 52547.09 | 5.3 | -0.088 | 92.85 | 45.91 | E.R., vacu, cyto |
| SlUGT71 | Solyc07g043100.1 | 7 | 468 | C _2381_ H _3728_ N _620_ O _696_ S _21_ | 52848.79 | 5.38 | -0.035 | 97.03 | 43.57 | cyto, chlo, nucl |
| SlUGT72 | Solyc07g043110.1 | 7 | 479 | C _2437_ H _3840_ N _640_ O _712_ S _20_ | 54138.36 | 5.54 | -0.085 | 98.25 | 42.25 | chlo, cyto, nucl |
| SlUGT73 | Solyc07g043120.1 | 7 | 490 | C _2472_ H _3861_ N _647_ O _722_ S _17_ | 54741.77 | 5.62 | -0.098 | 93.51 | 44.12 | chlo, cyto, nucl |
| SlUGT74 | Solyc07g043150.1 | 7 | 482 | C_2422_H_3770_N_632_O_711_S_24_ | 53887.83 | 5.52 | -0.096 | 85.93 | 43.77 | nucl, chlo, cyto |
| SlUGT75 | Solyc07g043160.1 | 7 | 440 | C_2240_H_3444_N_574_O_660_S_18_ | 49552.52 | 5.09 | -0.167 | 83.95 | 48.56 | chlo, vacu, nucl |
| SlUGT76 | Solyc07g043170.2 | 7 | 529 | C_2643_H_4154_N_676_O_804_S_25_ | 59065.6 | 4.89 | -0.098 | 95.09 | 50.12 | extr, vacu, E.R. |
| SlUGT77 | Solyc07g043190.1 | 7 | 292 | C _1501_ H _2323_ N _383_ O _421_ S _20_ | 33111.47 | 6.71 | -0.118 | 84.79 | 39.59 | chlo, mito, extr |
| SlUGT78 | Solyc07g043410.1 | 7 | 482 | C_2458_H_3813_N_637_O_692_S_27_ | 54225.79 | 5.8 | -0.011 | 94.07 | 42.27 | cyto, nucl, chlo |
| SlUGT79 | Solyc07g043480.1 | 7 | 456 | C_2283_H_3559_N_629_O_671_S_23_ | 51291.56 | 5.85 | -0.24 | 88.53 | 53.73 | nucl, cysk, cyto |
| SlUGT80 | Solyc07g043490.1 | 7 | 483 | C_2495_H_3846_N_642_O_715_S_19_ | 54885 | 5.55 | -0.165 | 91.64 | 48.3 | chlo, cyto, extr |
| SlUGT81 | Solyc07g043500.1 | 7 | 441 | C_2278_H_3551_N_591_O_658_S_17_ | 50290.84 | 5.7 | -0.245 | 92.54 | 38.85 | chlo, cyto, extr |
| SlUGT82 | Solyc08g006410.2 | 8 | 458 | C_2344_H_3694_N_600_O_686_S_22_ | 51962.04 | 6.25 | -0.247 | 91.68 | 48.49 | chlo, mito, nucl |
| SlUGT83 | Solyc08g014050.1 | 8 | 488 | C_2495_H_3845_N_645_O_715_S_18_ | 54893.95 | 5.5 | -0.131 | 92.4 | 42.11 | cyto, nucl, chlo |
| SlUGT84 | Solyc08g062220.2 | 8 | 460 | C_2326_H_3592_N_582_O_691_S_21_ | 51438.85 | 5.56 | -0.14 | 84.3 | 38.75 | chlo, nucl, extr |
| SlUGT85 | Solyc08g077080.1 | 8 | 481 | C_2443_H_3794_N_628_O_727_S_17_ | 54139.79 | 5.31 | -0.178 | 92.04 | 44.58 | chlo, vacu, nucl |
| SlUGT86 | Solyc09g008050.2 | 9 | 476 | C_2437_H_3788_N_630_O_706_S_13_ | 53625.46 | 5.87 | -0.054 | 101.51 | 31.95 | chlo, nucl, cyto |
| SlUGT87 | Solyc09g008060.2 | 9 | 500 | C_2569_H_3930_N_688_O_739_S_16_ | 56790.59 | 6.28 | -0.223 | 88.3 | 34.42 | chlo, nucl, cyto |
| SlUGT88 | Solyc09g008510.1 | 9 | 497 | C_2529_H_4007_N_659_O_742_S_30_ | 56478.4 | 5.75 | -0.143 | 95.67 | 45.56 | chlo, nucl, cysk |
| SlUGT89 | Solyc09g092480.1 | 9 | 468 | C_2364_H_3664_N_612_O_708_S_22_ | 52692.09 | 5.16 | -0.226 | 84.7 | 45.29 | nucl, cyto, chlo |
| SlUGT90 | Solyc09g092500.1 | 9 | 470 | C_2360_H_3705_N_617_O_715_S_18_ | 52739.16 | 5.19 | -0.281 | 88.13 | 52.14 | chlo, cyto, plas |
| SlUGT91 | Solyc09g098080.2 | 9 | 476 | C_2447_H_3796_N_622_O_713_S_19_ | 53945.94 | 5.62 | -0.108 | 92.12 | 40.23 | nucl, cyto, plas |
| SlUGT92 | Solyc10g008860.1 | 10 | 456 | C_2320_H_3601_N_605_O_667_S_23_ | 51378.15 | 6.13 | -0.223 | 83.57 | 33.62 | cyto, nucl, vacu |
| SlUGT93 | Solyc10g009580.2 | 10 | 476 | C_2406_H_3810_N_628_O_700_S_19_ | 53343.65 | 5.88 | -0.075 | 100.5 | 37.92 | cyto, E.R., nucl |
| SlUGT94 | Solyc10g079320.1 | 10 | 495 | C_2519_H_3924_N_700_O_730_S_15_ | 56176.02 | 9.21 | -0.365 | 80.69 | 45.13 | pero, chlo, mito |
| SlUGT95 | Solyc10g079330.1 | 10 | 436 | C_2233_H_3521_N_631_O_626_S_13_ | 49640.15 | 9.67 | -0.271 | 92.52 | 58.48 | chlo, extr |
| SlUGT96 | Solyc10g079340.1 | 10 | 433 | C_2196_H_3406_N_594_O_637_S_27_ | 49186.42 | 5.87 | -0.247 | 86.44 | 42.34 | nucl, cyto, chlo |
| SlUGT97 | Solyc10g079350.1 | 10 | 483 | C_2432_H_3774_N_666_O_720_S_22_ | 54468.07 | 5.65 | -0.296 | 85.18 | 46.23 | nucl, cyto, cysk |
| SlUGT98 | Solyc10g079930.1 | 10 | 437 | C_2217_H_3457_N_607_O_649_S_18_ | 49575.59 | 6.13 | -0.253 | 87.85 | 46.3 | chlo, chlo_mito, cyto |
| SlUGT99 | Solyc10g079950.1 | 10 | 486 | C_2449_H_3827_N_659_O_737_S_21_ | 54967.56 | 5.77 | -0.269 | 85.02 | 56.15 | nucl, cyto, cysk |
| SlUGT100 | Solyc10g079980.1 | 10 | 471 | C_2410_H_3807_N_653_O_693_S_21_ | 53690.96 | 8.98 | -0.244 | 90.36 | 48.04 | chlo, mito, extr |
| SlUGT101 | Solyc10g083440.1 | 10 | 447 | C_2243_H_3512_N_576_O_658_S_16_ | 49588.98 | 6.34 | -0.102 | 90.74 | 44.79 | chlo, nucl, mito |
| SlUGT102 | Solyc10g083860.1 | 10 | 494 | C_2440_H_3915_N_653_O_716_S_25_ | 54656.37 | 6.78 | -0.075 | 94.7 | 46.34 | chlo, vacu, nucl |
| SlUGT103 | Solyc10g085230.1 | 10 | 475 | C_2406_H_3763_N_625_O_697_S_29_ | 53526.85 | 5.33 | -0.012 | 96 | 55.94 | nucl, chlo, cyto |
| SlUGT104 | Solyc10g085240.1 | 10 | 471 | C_2395_H_3759_N_625_O_688_S_25_ | 53118.47 | 5.93 | -0.092 | 93.69 | 49.43 | chlo, nucl, cyto |
| SlUGT105 | Solyc10g085280.1 | 10 | 475 | C_2408_H_3780_N_634_O_694_S_26_ | 53549.89 | 5.53 | -0.006 | 99.31 | 58 | chlo, nucl, cyto |
| SlUGT106 | Solyc10g085860.1 | 10 | 494 | C_2497_H_3997_N_671_O_709_S_32_ | 55788.19 | 6.4 | -0.021 | 102.35 | 41.98 | extr, chlo, nucl |
| SlUGT107 | Solyc10g085870.1 | 10 | 486 | C_2461_H_3894_N_658_O_709_S_30_ | 55005.77 | 5.58 | -0.136 | 94.65 | 40.09 | chlo, nucl, cyto |
| SlUGT108 | Solyc10g085880.1 | 10 | 344 | C_1742_H_2764_N_476_O_527_S_22_ | 39513.3 | 5.19 | -0.465 | 82.44 | 46.45 | cyto, nucl, cysk |
| SlUGT109 | Solyc10g086240.1 | 10 | 498 | C_2557_H_3956_N_650_O_755_S_20_ | 56524.64 | 5.31 | -0.116 | 94.86 | 35.9 | chlo, nucl, cyto |
| SlUGT110 | Solyc11g006100.1 | 11 | 474 | C_2389_H_3745_N_647_O_704_S_17_ | 53339.95 | 6.02 | -0.185 | 94.81 | 45.82 | cyto, E.R._plas, plas |
| SlUGT111 | Solyc11g007350.1 | 11 | 463 | C_2465_H_3820_N_648_O_678_S_15_ | 53862.28 | 7.26 | -0.273 | 91.58 | 36.7 | cyto, nucl, E.R. |
| SlUGT112 | Solyc11g007370.1 | 11 | 444 | C_2289_H_3596_N_586_O_654_S_15_ | 50270.17 | 5.87 | -0.081 | 101.15 | 48.08 | chlo, cyto, extr |
| SlUGT113 | Solyc11g007380.1 | 11 | 447 | C_2294_H_3662_N_598_O_649_S_22_ | 50709.25 | 7.56 | -0.063 | 100.47 | 37.43 | chlo, mito, E.R. |
| SlUGT114 | Solyc11g007450.1 | 11 | 321 | C_1617_H_2574_N_434_O_491_S_11_ | 36303.5 | 5.03 | -0.354 | 92.59 | 40.64 | cyto, cyto_nucl, chlo |
| SlUGT115 | Solyc11g007460.1 | 11 | 436 | C_2242_H_3526_N_580_O_633_S_16_ | 49247.13 | 6.04 | -0.129 | 99.72 | 37.39 | chlo, cyto, mito |
| SlUGT116 | Solyc11g007470.1 | 11 | 442 | C_2260_H_3555_N_599_O_649_S_18_ | 50078.79 | 5.71 | -0.229 | 95.09 | 48.31 | chlo, cyto, mito |
| SlUGT117 | Solyc11g007480.1 | 11 | 453 | C_2314_H_3655_N_603_O_677_S_18_ | 51332.19 | 5.3 | -0.177 | 99.62 | 38.96 | chlo, cyto, mito |
| SlUGT118 | Solyc11g007490.1 | 11 | 443 | C_2263_H_3566_N_586_O_667_S_20_ | 50295.94 | 5.55 | -0.269 | 90.43 | 54.13 | cyto, chlo, nucl |
| SlUGT119 | Solyc11g010740.1 | 11 | 483 | C_2472_H_3857_N_637_O_714_S_17_ | 54469.68 | 5.48 | -0.131 | 96.65 | 37.75 | cyto, chlo, nucl |
| SlUGT120 | Solyc11g010760.1 | 11 | 474 | C_2438_H_3784_N_626_O_704_S_18_ | 53705.72 | 5.56 | -0.194 | 92.32 | 36.9 | cyto, nucl, chlo |
| SlUGT121 | Solyc11g010780.1 | 11 | 368 | C_1937_H_2965_N_479_O_567_S_9_ | 42323.26 | 4.87 | -0.243 | 87.64 | 42.08 | cyto, nucl, chlo |
| SlUGT122 | Solyc11g010810.1 | 11 | 470 | C_2515_H_3868_N_614_O_703_S_10_ | 54274.67 | 5.46 | -0.226 | 95.4 | 36.42 | chlo, cyto, nucl |
| SlUGT123 | Solyc11g061980.1 | 11 | 494 | C_2523_H_3890_N_656_O_736_S_17_ | 55733.61 | 5.39 | -0.205 | 88.81 | 36.75 | cyto, nucl, chlo |
| SlUGT124 | Solyc11g066670.1 | 11 | 467 | C_2329_H_3637_N_635_O_677_S_18_ | 51942.43 | 5.9 | -0.133 | 88.07 | 49.36 | cyto, cysk, nucl |
| SlUGT125 | Solyc11g066680.1 | 11 | 464 | C_2321_H_3637_N_631_O_684_S_20_ | 51966.43 | 5.9 | -0.211 | 86.31 | 53.63 | chlo, cyto, mito |
| SlUGT126 | Solyc12g006430.1 | 12 | 442 | C_2243_H_3538_N_598_O_638_S_30_ | 50052.19 | 7.92 | -0.105 | 92.83 | 40.15 | chlo, E.R., nucl |
| SlUGT127 | Solyc12g009910.1 | 12 | 420 | C_2100_H_3325_N_593_O_629_S_18_ | 47521.18 | 6.36 | -0.228 | 92.07 | 44.23 | chlo, nucl, cyto |
| SlUGT128 | Solyc12g009930.1 | 12 | 412 | C_2083_H_3293_N_555_O_627_S_19_ | 46752.54 | 5.52 | -0.238 | 91.99 | 42.84 | nucl, cyto, cysk |
| SlUGT129 | Solyc12g009940.1 | 12 | 460 | C_2317_H_3624_N_624_O_694_S_25_ | 52127.53 | 5.18 | -0.205 | 87.46 | 43.56 | cyto, cysk, nucl |
| SlUGT130 | Solyc12g014010.1 | 12 | 483 | C_2422_H_3804_N_618_O_720_S_23_ | 53837.94 | 5.38 | -0.136 | 88.41 | 46.64 | E.R._plas, plas, E.R. |
| SlUGT131 | Solyc12g042600.1 | 12 | 494 | C_2479_H_3961_N_669_O_714_S_29_ | 55491.51 | 6.26 | -0.151 | 95.85 | 40.55 | cyto, nucl, cysk |
| SlUGT132 | Solyc12g057060.1 | 12 | 486 | C_2445_H_3795_N_631_O_728_S_23_ | 54415.2 | 5.33 | -0.212 | 86.03 | 47.01 | chlo, cyto, mito |
| SlUGT133 | Solyc12g057080.1 | 12 | 486 | C_2448_H_3811_N_633_O_728_S_24_ | 54527.43 | 5.28 | -0.19 | 88.23 | 39.23 | chlo, cyto, nucl |
| SlUGT134 | Solyc12g088690.1 | 12 | 319 | C_1610_H_2538_N_424_O_481_S_18_ | 36107.5 | 5.8 | -0.208 | 87.37 | 41.82 | nucl, cyto, cysk |
| SlUGT135 | Solyc12g088700.1 | 12 | 396 | C_2028_H_3157_N_529_O_593_S_24_ | 45207 | 6.05 | -0.298 | 81.46 | 53.34 | nucl, cyto, chlo |
| SlUGT136 | Solyc12g088710.1 | 12 | 485 | C_2481_H_3833_N_643_O_730_S_24_ | 55118.04 | 5.37 | -0.2 | 87.79 | 47.47 | nucl, cyto, cysk |
| SlUGT137 | Solyc12g096080.1 | 12 | 486 | C_2521_H_3924_N_646_O_715_S_18_ | 55299.87 | 8.29 | -0.227 | 91.42 | 43.08 | nucl, chlo, cyto |
| SlUGT138 | Solyc12g096820.1 | 12 | 464 | C_2386_H_3735_N_599_O_694_S_23_ | 52653.88 | 5.47 | -0.153 | 92.13 | 45.83 | chlo, nucl, plas |
| SlUGT139 | Solyc12g096830.1 | 12 | 453 | C_2329_H_3647_N_591_O_680_S_24_ | 51576.57 | 5.54 | -0.143 | 91.99 | 45.73 | nucl, chlo, cyto |
| SlUGT140 | Solyc12g096870.1 | 12 | 486 | C_2459_H_3837_N_649_O_722_S_26_ | 54877.99 | 5.43 | -0.174 | 88 | 56 | chlo, nucl, mito |
| SlUGT141 | Solyc12g098580.1 | 12 | 465 | C_2352_H_3682_N_624_O_693_S_20_ | 52430.07 | 5.9 | -0.218 | 91.96 | 41.11 | chlo, vacu, nucl |
| SlUGT142 | Solyc12g098590.1 | 12 | 470 | C_2379_H_3689_N_613_O_700_S_22_ | 52783.47 | 5.38 | -0.25 | 86.89 | 48.62 | chlo, cyto |
| SlUGT143 | Solyc12g098600.1 | 12 | 463 | C_2323_H_3614_N_610_O_695_S_20_ | 51849.12 | 5.17 | -0.151 | 90.52 | 39.97 | nucl, cyto, chlo |

**Table S2 Primers used in this study**

| Primer name | Primer sequence |
| --- | --- |
| P450F | TACTTCCTTCGCGGATCGAC |
| P450R | TCGCCTTACTCGCACTGTTT |
| GSTF | TGGTGCAGCACTTTATTTGGG |
| GSTR | ACAGGCTGAATGTAGGCTCG |
| ABCF | AGGGATTTCAGGGGGACAGA |
| ABCR | CCCTTGGTGCACCCATTAGT |
| SlUGT9F | AAGCAGGATGAGCAAAGGCT |
| SlUGT9R | ACCTTCACACCATGCCTAGC |
| SlUGT11F | GAAGCTTGTCGCTTGTCGTG |
| SlUGT11R | CTTCGCATCCGATTCCCTCA |
| SlUGT26F | TCAGATCGCGATGCAACTCA |
| SlUGT26R | CTCAGCAAAGAATGGCCAGC |
| SlUGT33F | GTTGAGGGACGCGGATAGAG |
| SlUGT33R | ACAAGTAGCCAGCAATGTTCT |
| SlUGT84F | CCATTTCCGTGAGCCAGTCT |
| SlUGT84R | TCCAACTGAGGTGCCCAATC |
| SlUGT101F | GCGCGAAGCAATTGAACTCT |
| SlUGT101R | CGAGTTCCATCCACAATGCG |
| SlUGT108F | TGGAAGGTATAACCGCTGGC |
| SlUGT108R | GCCTCTCTATGCCTGACCTC |
| SlUGT131F | AGAGGCAGGGTTACCACAAG |
| SlUGT131R | TGAGCCTCTCCCTCCTTCAT |
